# The effect of root exudates on rhizosphere water dynamics

**DOI:** 10.1098/rspa.2018.0149

**Published:** 2018-09-05

**Authors:** L. J. Cooper, K. R. Daly, P. D. Hallett, N. Koebernick, T. S. George, T. Roose

**Affiliations:** 1 Bioengineering Sciences Research Group, Faculty of Engineering and the Environment, University of Southampton, Southampton, UK; 2 School of Biological Sciences, University of Aberdeen, Aberdeen, UK; 3 The James Hutton Institute, Invergowrie, Dundee, UK

**Keywords:** homogenization, porous media, Richards' equation

## Abstract

Most water and nutrients essential for plant growth travel across a thin zone of soil at the interface between roots and soil, termed the rhizosphere. Chemicals exuded by plant roots can alter the fluid properties, such as viscosity, of the water phase, potentially with impacts on plant productivity and stress tolerance. In this paper, we study the effects of plant exudates on the macroscale properties of water movement in soil. Our starting point is a microscale description of two fluid flow and exudate diffusion in a periodic geometry composed from a regular repetition of a unit cell. Using multiscale homogenization theory, we derive a coupled set of equations that describe the movement of air and water, and the diffusion of plant exudates on the macroscale. These equations are parametrized by a set of cell problems that capture the flow behaviour. The mathematical steps are validated by comparing the resulting homogenized equations to the original pore scale equations, and we show that the difference between the two models is ≲7% for eight cells. The resulting equations provide a computationally efficient method to study plant–soil interactions. This will increase our ability to predict how contrasting root exudation patterns may influence crop uptake of water and nutrients.

## 1. Introduction

Jethro Tull's [1] 1762 observation that ‘roots are but as guts inverted… that spew out what is superfluous’ recognized the capacity of plants to exude chemicals from their roots to capture nutrients from the soil. More recent research has observed that these surface active chemicals alter fluid properties at the root–soil interface considerably [2]. This has the potential to affect storage and transport of water and nutrients. Plant breeding may be able to manipulate the chemistry and quantity of these exudates to improve resource capture and stress tolerance from droughts and floods, potentially addressing food security by improving yield. Certainly between species of plants, root exudate properties vary considerably. Understanding of root exudates and root–soil interactions can lead to advances in agricultural techniques to improve food production, particularly in extreme conditions [3]. Mathematical modelling provides one route through which the complexities of water and nutrient movement in soils can be understood [4]. Hence, developing new mathematical models, which use the vast computational resources that are now available, will lead to a significant improvement in root–soil interaction models. This in turn will further improve, and potentially optimize, crop yield.

Richards' equation is widely used to model the movement of water through partially saturated porous media, including soil, at large scales [5]. Traditionally, Richards' equation is derived by combining conservation of mass with Darcy's Law and parametrized by equilibrium measurements of the soil water characteristic curve (SWCC) and hydraulic conductivity [6,7]. More recently, Daly & Roose [8] used the Cahn–Hilliard–Stokes equations, which have been used to model two fluid flow in porous media [9–11], to show that these hydraulic properties of a partially saturated soil can be evaluated from the underlying geometry.

The equations derived by Daly & Roose [8] are appropriate for modelling bulk soil. However, they might not be directly applicable to the region of soil close to the roots over which the plants have influence, known as the rhizosphere [12]. The rhizosphere can have different structural, chemical, biological and hydraulic properties to the bulk soil [13–15]. This can be partially due to the presence of root exudates. Root exudates mix with soil pore water, creating a diffusion gradient away from the surface of the root. When exudates mix with soil pore water they can decrease the surface tension and soil–water content, increase viscosity and affect the contact angle between soil particles and pore water [2,16–18]. These impacts, however, vary between species of plants and it may be possible to breed future crops that produce exudates with desired physical impacts on soil pore water.

The impact of the altered hydraulic properties on the movement of water at large scales has been investigated, but is not well understood [13,15]. Raynaud [19] used Fickian diffusion to model exudates from a root in a simple cylindrical geometry. The water content was assumed constant and the water movement was controlled by the rate of uptake by the root, a sink term was used to model exudate decay and a source term was present at the root surface to model exudation from the root. The gradient of the adsorption isotherm, decay rate of the exudate, and soil water content primarily determined the time for the concentration to reach steady state. The effects of exudates on the macroscale have been considered in conditions where the viscosity dominates over surface tension in regions of high root exudate content [15,20]. They found that an increase in hydraulic connectivity of the rhizosphere due to the formation of liquid bridges. Daly *et al.* [13] used the model derived in Daly & Roose [8] to study the effect of increased contact angle, surface tension and viscosity. However, they did not explicitly model exudate diffusion or how this would affect the derivation of Richards' equation.

The work presented here is motivated by the effect of root exudates on soil, however, the theory can also be applied to areas such as geological waste disposal, oil production or oil-spill clean-up problems. Numerical methods have been used to investigate two fluid flow with mass transfer on the pore scale for applications in chemical engineering, such as determining the rate of CO_2_ capture [21,22]. Yang *et al.* [21] and Haroun *et al.* [22] implemented the Navier–Stokes equations, using the one fluid formulation with a characteristic function to define the interface, and are coupled to the mass transfer equation through the local velocity. In these studies, the solute concentration does not affect the behaviour of the fluid flow and the solute is able to diffuse across the interface between the two fluids, which have different diffusion coefficients [21,23,24]. Davidson & Rudman [23] considered a solute within a spherical drop of one fluid containing a solute within a second fluid. They validated the numerical calculations by comparison with the analytical solutions and considered mass transfer of a solute from a drop rising in a fluid column. Haroun *et al.* [24] examined the effect of a periodic corrugated geometry on mass transfer and found that recirculation zones, which held up the movement of the liquid, affected the mass transfer because it changed the shape of the fluid–fluid interface. Yang *et al.* [21] created a model of a microscale segmented flow microreactor in OpenFOAM, which shows the gas transfer between a gas and liquid phase, and could be used to optimize this type of system.

In this paper, we derive macroscale models for water movement in soil that take account of changes to fluid properties due to the presence of root exudates and the underlying pore scale geometry. To do this, we have extended the derivation of Daly & Roose [8] by developing a pore scale description of exudate diffusion, which we have coupled to a two fluid model for water movement. By including coupling terms to link the fluid properties to the exudate diffusion we were able to capture the effect of exudates on hydraulic properties. We have applied homogenization theory [25] to upscale the model from the pore scale to the macroscale, e.g. pot or field scale, and have obtained a set of coupled equations for water movement and the diffusion of exudates. The upscaling procedure used to develop the macroscale model has been validated against the underlying pore scale equations using an idealized geometry. The upscaled equations agree with the underlying pore scale equations within less than 7% error.

## 2. Derivation of equations

In this section, we describe the derivation of the macroscale coupled flow and diffusion equations. Our aim is to start with a set of equations on the pore scale and to use these to derive a set of macroscale equations. We will start with the Cahn–Hilliard two fluid model and couple this to a phase-dependent diffusion equation, which describes the movement of root exudates through the pore water.

### (a) The pore scale model

**Figure 1.**
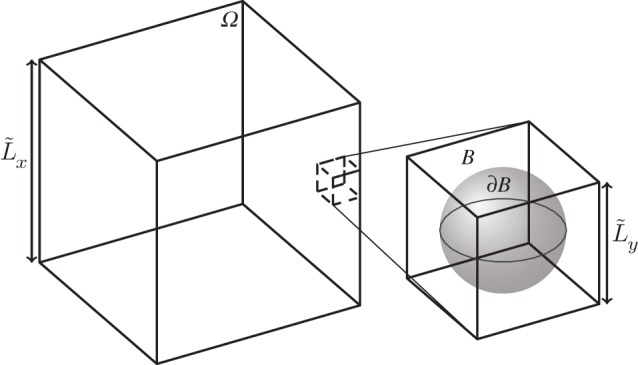
The macroscale soil domain, *Ω*, and one of the periodic units. An idealized porescale periodic unit contains a fluid domain, *B*, and an illustrative example of a soil particle surface, ∂*B*, shaded in grey.

We consider a macroscale soil domain, *Ω*. On the pore scale, this is composed of repeating periodic units. The periodic units contain a fluid domain, *B*, and the soil particle surface, ∂*B*, as illustrated in figure 1. We start with the dimensional Cahn–Hilliard–Stokes equations, which we write following the notation used in Daly & Roose [8]:

2.1*a* $$\begin{array}{rl} & \frac{\partial \phi }{\partial \tilde{t}}+\tilde{\nabla \;\nabla }\cdot (\phi \tilde{u\;u})=\tilde{\nabla \;\nabla }\cdot \frac{\phi^{2}{\left(1-\phi \right)}^{2}}{\tilde{\zeta }}\tilde{\nabla \;\nabla }\tilde{\mu },\;\tilde{x}\in B, \end{array}$$

2.1*b* $$\begin{array}{rl} & \tilde{\nabla \;\nabla }\cdot \tilde{\eta }\tilde{\sigma \;\sigma }-\tilde{\nabla \;\nabla }\tilde{p}-\phi \tilde{\nabla \;\nabla }\tilde{\mu }=\tilde{\rho }\tilde{g}{\hat{e\;e}}_{3},\;\;\;\tilde{x}\in B \end{array}$$

2.1*c* $$\begin{array}{rl} \;\text{and}\; & \tilde{\nabla \;\nabla }\cdot \tilde{u\;u}=0,\;\;\;\;\;\;\;\tilde{x}\in B. \end{array}$$

Here, the notation $\tilde{\cdot }$ denotes a dimensional value, *ϕ* is the dimensionless fluid phase field, which takes the value *ϕ* = 1 in water and *ϕ* = 0 in air, $\tilde{u\;u}=\phi {\tilde{u\;u}}^{\left(w\right)}+(1-\phi ){\tilde{u\;u}}^{\left(a\right)}$ is the combined velocity, where ${\tilde{u\;u}}^{\left(w\right)}$ and ${\tilde{u\;u}}^{\left(a\right)}$ are the water and air velocities, respectively, and $\tilde{\sigma \;\sigma }=(\tilde{\nabla \;\nabla }\tilde{u\;u})+{\left(\tilde{\nabla \;\nabla }\tilde{u\;u}\right)}^{T}$ is the viscous stress tensor. $\tilde{\rho }(\phi )$ is the phase-dependent density, which takes the value of the density of air when *ϕ* = 0 and takes the value of the density of water when *ϕ* = 1, $\tilde{\eta }(\phi )$ is the phase-dependent viscosity, $\tilde{g}$ is the acceleration due to gravity, $\tilde{t}$ is time, $\tilde{\zeta }$ is the drag coefficient between the water and air, and $\tilde{p}$ is the combined pressure that enforces incompressibility of both the water and air phases. The mobility is generally free to choose, up to some structural requirements for tensors, and we have used $\tilde{M}=\phi^{2}{\left(1-\phi \right)}^{2}/\tilde{\zeta }$ for consistency with the homogenization of two fluids literature [8]. We note that in the case of Daly & Roose [8], it was derived directly from a free energy based on the idea that the two fluids exert a drag on each other with drag coefficient $\tilde{\zeta }$. The capillary pressure, $\tilde{\mu }$, is given by

2.2 $$\tilde{\mu }=\tilde{\gamma }\alpha ({\tilde{\lambda }}^{-1}f^{\prime }(\phi )-\tilde{\lambda }{\tilde{\nabla }}^{2}\phi ),\;\tilde{x}\in B,$$

where $\tilde{\gamma }$ is the surface tension between air and water, $\alpha =6\sqrt{2}$, $\tilde{\lambda }$ is the air–water interface thickness, which is small compared with the geometry on which the model is applied, and *f*(*ϕ*) = *ϕ*^2^(1 − *ϕ*)^2^ is the energy of the two fluid system. *f*(*ϕ*) is chosen to have minima at *ϕ* = 0 and *ϕ* = 1. Hence, equation (2.2) has the solution $\phi =0+O(\tilde{\lambda })$ and $\phi =1+O(\tilde{\lambda })$ everywhere except for a region of width $\tilde{\lambda }$, where *ϕ* varies rapidly.

Equations (2.1) and (2.2) are the Cahn–Hilliard–Stokes equations, where equation (2.1a) describes the movement of the air and water phases driven by the velocity of the combined velocity, $\tilde{u\;u}$, and the capillary pressure, $\tilde{\mu }$. Equation (2.1b) is Stokes equation with capillary pressure and gravitational effects. Equation (2.1c) ensures that the mass of both the water and air is conserved. The Cahn–Hilliard model has been used here as it has previously been homogenized allowing us to build on existing theory [8,26–28]. Here we extend from the previous application to a fuel cell in [26,27] to consider fluid flow in soil. Using a phase field variable within the Cahn–Hilliard framework allows the model to be more general than other models such as Korteweg theory, which is restricted to using density as the order parameter [29].

In order to be able to homogenize the equations, it is required that the component of velocity normal to the soil surface is zero, i.e. a zero penetration condition must be used. This can be combined with a no-slip, or generalized Navier slip condition. Here, we demonstrate the method using a no-slip condition on the soil boundary, ∂*B*, i.e.

2.3*a* $$\tilde{u\;u}=0,\;\tilde{x}\in \partial B.$$

A flux condition that defines the behaviour between the water–air interface and the soil particle surface using the soil–liquid contact angle *θ*, as in Daly *et al.* [13], is given by

2.3*b* $$\hat{n\;n}\cdot \tilde{\nabla \;\nabla }\phi =|\tilde{\nabla \;\nabla }\phi |\cos(\tilde{\theta }(\tilde{c})),\;\tilde{x}\in \partial B,$$

and a zero flux condition for the capillary pressure is given by

2.3*c* $$\hat{n\;n}\cdot \frac{\phi^{2}{\left(1-\phi \right)}^{2}}{\tilde{\zeta }}\tilde{\nabla \;\nabla }\tilde{\mu }=0,\;\tilde{x}\in \partial B.$$

The initial saturation $\bar{S}$ is chosen and used to establish the initial condition for the phase, $\bar{\phi }$, i.e. $\phi (\tilde{x},0)=\bar{\phi }(\bar{S})$, where

2.4 $$\bar{S}=\frac{1}{∥B∥}\int_{B}\phi \;dy\;y,$$

where ∥*B*∥ is the volume of the pore scale domain. The initial velocity is defined as ***u*** = 0.

Assuming the functions $\tilde{\gamma }$, $\tilde{\eta }$ and $\tilde{\theta }$ are specified, e.g. from experiments, equations (2.1), (2.2) and (2.3) provide a complete description of the fluid velocity, pressure and phase on the pore scale, respectively. From new experimental evidence [2], we will assume that certain features of the fluid model are dependent on the concentration of root exudate. In keeping with the literature, we assume that the root exudate concentration $\tilde{c}$ affects the air–water contact angle at the surface of the porous material, $\theta (\tilde{c})$, surface tension, $\tilde{\gamma }(\tilde{c})$, viscosity, $\tilde{\eta }(\phi ,c)$ [13,14,30] and the fluid–fluid drag coefficient $\tilde{\zeta }(c)$. The functions $\theta (\tilde{c})$, $\tilde{\gamma }(\tilde{c})$ and $\tilde{\eta }(\phi ,c)$ can be parametrized using experimental measurements [2]. The concentration-dependent functions are expected to depend nonlinearly on the concentration of root exudate. Fitting to the experimental data from [2] shows that this dependence is quadratic. The fluid–fluid drag coefficient is particularly difficult to parametrize, and it is assumed to be linearly dependent on the viscosity $\tilde{\zeta }=\tilde{\varsigma }\tilde{\eta }(\phi ,c)$, where $\tilde{\varsigma }$ is a constant with units m^−2^. This implies that the greater the viscosity of the water phase, the greater the drag between the water and air phases.

The viscosities of the water and air are combined into one function using *ϕ*. The viscosity, $\tilde{\eta }(c,\phi )$, takes the value, ${\tilde{\eta }}^{\left(a\right)}$ when *ϕ* = 0, and ${\tilde{\eta }}^{\left(w\right)}(c)$ when *ϕ* = 1. Here, ${\tilde{\eta }}^{\left(a\right)}$ is the viscosity of air and ${\tilde{\eta }}^{\left(w\right)}(c)$ is the viscosity of water, which depends on the concentration of root exudate. The viscosity function is defined as

2.5 $$\tilde{\eta }(\phi ,c)={\tilde{\eta }}^{\left(a\right)}+[{\tilde{\eta }}^{\left(w\right)}(c)-{\tilde{\eta }}^{\left(a\right)}]\phi .$$

In order to couple the Cahn–Hilliard–Stokes equations with the diffusion of root exudates through water, we introduce a phase-dependent diffusion equation. We assume that the exudate can diffuse in water with a diffusion constant, $\tilde{D}\neq 0$, and that in the air-filled pore space the exudate is immobile. As the interface is mobile, an additional term needs to be included to ensure the exudate is advected with the interface as it moves. This is achieved by adding an advection term to the diffusion model, which accounts for the movement of the phase, *ϕ*. From a mathematical point of view, this term ensures that the concentration in the water phase is conserved and decays with the phase field at the air–water interface. Hence, the phase-dependent concentration equation is

2.6*a* $$\frac{\partial \tilde{c}}{\partial \tilde{t}}+\tilde{u\;u}\cdot \tilde{\nabla \;\nabla }\tilde{c}=\tilde{\nabla \;\nabla }\cdot \tilde{D}(\phi \tilde{\nabla \;\nabla }\tilde{c}-\tilde{c}\tilde{\nabla \;\nabla }\phi ),\;\tilde{x}\in B$$

and

2.6*b* $$\hat{n\;n}\cdot \tilde{D}(\phi \tilde{\nabla \;\nabla }\tilde{c}-\tilde{c}\tilde{\nabla \;\nabla }\phi )=0,\;\;\;\;\tilde{x}\in \partial B.$$

Equations (2.6) are similar to the equation used by Haroun *et al.* [24,31] without the expression of Henry's Law which allows the chemical species to diffuse across the interface.

### (b) Dimensionless equations

Equations (2.1)–(2.6) describe the fluid flow in soil coupled with root exudate diffusion. Equations (2.1)–(2.6) are non-dimensionalized using $\tilde{x\;x}={\tilde{L}}_{y}y\;y$, where ${\tilde{L}}_{y}$ is the microscopic length scale and $\nabla \;\nabla ={\tilde{L}}_{y}\tilde{\nabla \;\nabla }$, so the unit cell, *Y* = (0, 1)^3^, is defined with fluid part *B* and solid boundary ∂*B* [8]. The macroscopic length scale is ${\tilde{L}}_{x}$ and ${\tilde{L}}_{y}/{\tilde{L}}_{x}=\varepsilon \ll 1$. We assume the non-dimensional surface tension to be $\tilde{\gamma }=\bar{\gamma }\gamma (c)$, where $\bar{\gamma }$ is a baseline surface tension, e.g. the surface tension of water in air at 20°C, 0.072 N m^−1^, and *γ*(*c*) is the dimensionless exudate concentration-specific surface tension. We choose $\lambda =\tilde{\lambda }/{\tilde{L}}_{y}$. The non-dimensional variables, denoted without $\tilde{\cdot }$, are given by

2.7*a* $$\mu =\tilde{\mu }\frac{{\tilde{L}}_{y}}{\bar{\gamma }\alpha },\;p=\tilde{p}\frac{{\tilde{L}}_{y}}{\bar{\gamma }\alpha },\;u\;u=\tilde{u\;u}\frac{{\tilde{L}}_{x}{\tilde{\eta }}^{\left(w\right)}}{{\tilde{L}}_{y}\bar{\gamma }\alpha },\;t=\tilde{t}\frac{\bar{\gamma }\alpha }{{\tilde{L}}_{x}{\tilde{\eta }}^{\left(w\right)}},\;c=\tilde{c}\tilde{C},\;D=\tilde{D}\frac{{\tilde{\eta }}^{\left(w\right)}}{{\tilde{L}}_{y}\bar{\gamma }\alpha },$$

with

2.7*b* $$\eta (\phi ,c)=\frac{\tilde{\eta }(\phi ,c)}{{\tilde{\eta }}^{\left(w\right)}}=\frac{{\tilde{\eta }}^{\left(a\right)}}{{\tilde{\eta }}^{\left(w\right)}}+\frac{\left(\tilde{\eta }(c)-{\tilde{\eta }}^{\left(a\right)}\right)}{{\tilde{\eta }}^{\left(w\right)}}\phi \;\mathrm{and}\;\phi g=\frac{{\tilde{L}}_{x}{\tilde{L}}_{y}\tilde{g}}{\bar{\gamma }\alpha }\tilde{\rho }(\phi ),$$

where $g=({\tilde{\rho }}^{\left(w\right)}-{\tilde{\rho }}^{\left(a\right)})/{\tilde{\rho }}^{\left(w\right)}$ and *M* = *ϕ*^2^(1 − *ϕ*)^2^. We define the dimensionless drag/interface parameter $1/{\tilde{L}}_{y}^{2}\tilde{\varsigma }=\Upsilon$. Using these scalings, the non-dimensional Cahn–Hilliard, Stokes and phase-dependent advection–diffusion equations become

2.8*a* $$\begin{array}{rl} & \frac{\partial \phi }{\partial t}+u\;u\cdot \nabla \;\nabla \phi =\frac{\Upsilon }{\varepsilon }\nabla \;\nabla \cdot \frac{M}{\eta (c)}\nabla \;\nabla \mu ,\;y\;y\in B, \end{array}$$

2.8*b* $$\begin{array}{rl} & \nabla \;\nabla \cdot \eta (\phi ,c)\sigma \;\sigma -\frac{1}{\varepsilon }\nabla \;\nabla p-\frac{1}{\varepsilon }\phi \nabla \;\nabla \mu =\phi g{\hat{e\;e}}_{3},\;y\;y\in B, \end{array}$$

2.8*c* $$\begin{array}{rl} & \nabla \;\nabla \cdot u\;u=0,\;y\;y\in B \end{array}$$

2.8*d* $$\begin{array}{rl} \;\text{and}\; & \mu =\gamma (c)(\lambda^{-1}f^{\prime }(\phi )-\lambda {\nabla \;\nabla }^{2}\phi ),\;y\;y\in B, \end{array}$$

with boundary conditions

2.8*e* $$u\;u=0,\;\hat{n\;n}\cdot \nabla \;\nabla \phi =|\nabla \;\nabla \phi |\cos(\theta (c)),\;y\;y\in \partial B$$

and

2.8*f* $$\hat{n\;n}\cdot \frac{M}{\eta (c)}\nabla \;\nabla \mu =0,\;y\;y\in \partial B,$$

and the transport equation

2.8*g* $$\frac{\partial c}{\partial t}+u\;u\cdot \nabla \;\nabla c-\nabla \;\nabla \cdot \frac{D}{\varepsilon }(\phi \nabla \;\nabla c-c\nabla \;\nabla \phi )=0,\;y\;y\in B,$$

with boundary condition

2.8*h* $$\hat{n\;n}\cdot D(\phi \nabla \;\nabla c-c\nabla \;\nabla \phi )=0,\;y\;y\in \partial B,$$

where $\sigma \;\sigma \;=\;\nabla \;\nabla u\;u\;+\;{\left(\nabla \;\nabla u\;u\right)}^{T}$, and *D* is assumed to be constant. Here, to simplify the analysis, we have neglected the influence of gravity on the air phase, i.e. ${\tilde{\rho }}^{\left(a\right)}/{\tilde{\rho }}^{\left(w\right)}≲O(\varepsilon )$ [8]. The scalings used here have been chosen so that Υ∼O(1) so that the only small parameters are *ε* and λ. The scaling results in a unit change in *μ* driving a fluid velocity of order *ε*^−1^ that corresponds to the fastest time scale, which is defined in the next section, and implies that the capillary forces dominate as we have assumed. Therefore, the velocity and gravity contributions first appear at order 1, corresponding to the intermediate timescale.

### (c) Homogenization

The full set of equations (2.8) is fourth order, stiff and nonlinear. Hence, it is time consuming and computationally expensive to solve them numerically on real soil geometries. To overcome this issue, we derive a set of equations that describe how the pore scale dynamics affect the macroscale behaviour using the method of multiple scale asymptotic homogenization, an averaging procedure for periodic structures [25]. Homogenization requires two assumptions: firstly, that the macro and micro length scales, ${\tilde{L}}_{x}$ and ${\tilde{L}}_{y}$, can be treated independently; and secondly, that the underlying geometry is periodic on the pore scale.

Homogenization is based on a linear expansion of the dimensionless equations in terms of *ε*. This leads to a cascade of numerical problems, in which the details of the pore scale geometry are captured by solving representative cell problems on one period of the domain [25]. The results from the cell problems then parametrize averaged macroscale equations that approximate the solution of the full set of equations. Importantly, the homogenized equations are solved on an averaged geometry, i.e. they only depend on the pore scale geometry through the averaged parameters. This makes the macroscale equations much more efficient to solve than the original full set of equations.

In the homogenization presented here, there are some differences to the standard procedure. Firstly, in addition to the macro and micro length scales, we also consider three different time scales. The fastest time scale, *τ*_−1_, is associated with the leading-order pore scale dynamics, i.e. the equilibration of the air–water interface. The second time scale, *τ*_0_, is the intermediate timescale associated with fluid flow driven by flux imbalances on the pore scale. Finally, the slowest time scale, *τ*_1_, is associated with the macroscale behaviour, i.e. the slow variation in saturation due to macroscale pressure gradients. In addition, equations (2.8) are nonlinear and therefore the accuracy of the final macroscale approximation will depend on how well the equations can be approximated by linear expansions.

The homogenization procedure involves a large number of mathematical steps, which are somewhat analogous to the steps taken by Daly & Roose [8]. Hence, the full procedure has been detailed in the electronic supplementary material S1 and we have included here only the main steps and key results. We seek a perturbation expansion solution to equations (2.8), i.e. we use $\nabla \;\nabla \;=\;{\nabla \;\nabla }_{y}\;+\;\varepsilon {\nabla \;\nabla }_{x}$ and $t=\varepsilon^{-1}\tau_{1}+\tau_{0}+\varepsilon \tau_{-1}+O(\varepsilon^{2})$ with the expansions

2.9 $$\begin{array}{rl} \phi & =\sum_{k=0}^{\infty }\varepsilon^{k}\phi_{k}(y\;y,x\;x,\tau_{1},\tau_{0},\tau_{-1}),\;u\;u=\sum_{k=0}^{\infty }\varepsilon^{k}u\;u_{k}(y\;y,x\;x,\tau_{1},\tau_{0},\tau_{-1}),\\ M & =\sum_{k=0}^{\infty }\varepsilon^{k}M_{k}(y\;y,x\;x,\tau_{1},\tau_{0},\tau_{-1}),\;c=\sum_{k=0}^{\infty }\varepsilon^{k}c_{k}(y\;y,x\;x,\tau_{1},\tau_{0},\tau_{-1}),\\ \mu & =\sum_{k=0}^{\infty }\varepsilon^{k}\mu_{k}(y\;y,x\;x,\tau_{1},\tau_{0},\tau_{-1}),\;\sigma =\sum_{k=0}^{\infty }\varepsilon^{k}\sigma_{ky}(y\;y,x\;x,\tau_{1},\tau_{0},\tau_{-1})\\ p & =\sum_{k=0}^{\infty }\varepsilon^{k}p_{k}(y\;y,x\;x,\tau_{1},\tau_{0},\tau_{-1}),\;\mathrm{and}\;\sigma_{0y}=({\nabla \;\nabla }_{y}u\;u_{0})+{\left({\nabla \;\nabla }_{y}u\;u_{0}\right)}^{T}. \end{array}\}$$

Using Taylor series expansions, we also write, $1/\varepsilon \eta (c)=1/\varepsilon \eta_{0}-\eta_{1}/\eta_{0}^{2}+O(\varepsilon )$, where *η*_1_ = *c*_1_(*δη*/*δc*)|_*c*=*c*_0__, *M*_0_ = *ϕ*^2^_0_(1 − *ϕ*_0_)^2^, *M*_1_ = *ϕ*_1_(*δM*/*δϕ*)|_*ϕ*=*ϕ*_0__ and *δ*/*δv* is the functional derivative with respect to a function *v*. We substitute these expansions into equations (2.8) and solve the problems for increasing orders in *ε*.

#### (i) $O(\varepsilon^{-1})$

The first step in the homogenization procedure is to collect the dominant terms from equations (2.8) using expansions (2.9). In this case, the largest terms are of size *ε*^−1^. By collecting order *ε*^−1^ terms, we find

2.10*a* $$\begin{array}{rl} & \frac{\partial \phi_{0}}{\partial \tau_{-1}}=\Upsilon {\nabla \;\nabla }_{y}\cdot \frac{M_{0}}{\eta_{0}}{\nabla \;\nabla }_{y}\mu_{0},\;y\;y\in B, \end{array}$$

2.10*b* $$\begin{array}{rl} & -{\nabla \;\nabla }_{y}p_{0}-\phi_{0}{\nabla \;\nabla }_{y}\mu_{0}=0,\;y\;y\in B, \end{array}$$

2.10*c* $$\begin{array}{rl} & \mu_{0}=\gamma (c_{0})(\lambda^{-1}f^{\prime }(\phi_{0})-\lambda {\nabla \;\nabla }_{y}^{2}\phi_{0}),\;y\;y\in B \end{array}$$

2.10*d* $$\begin{array}{rl} \;\text{and}\; & \frac{\partial c_{0}}{\partial \tau_{-1}}-{\nabla \;\nabla }_{y}\cdot D(\phi_{0}{\nabla \;\nabla }_{y}c_{0}-c_{0}{\nabla \;\nabla }_{y}\phi_{0})=0,\;y\;y\in B, \end{array}$$

with boundary conditions,

2.10*e* $$\begin{array}{rl} & \hat{n\;n}\cdot {\nabla \;\nabla }_{y}\phi_{0}=|{\nabla \;\nabla }_{y}\phi_{0}|\cos(\theta (c_{0})),\;y\;y\in \partial B, \end{array}$$

2.10*f* $$\begin{array}{rl} & \hat{n\;n}\cdot \frac{M_{0}}{\eta_{0}}{\nabla \;\nabla }_{y}\mu_{0}=0,\;y\;y\in \partial B \end{array}$$

2.10*g* $$\begin{array}{rl} \;\text{and}\; & \hat{n\;n}\cdot D(\phi_{0}{\nabla \;\nabla }_{y}c_{0}-c_{0}{\nabla \;\nabla }_{y}\phi_{0})=0,\;y\;y\in \partial B, \end{array}$$

where *p*_0_, *μ*_0_, *ϕ*_0_ and *c*_0_ are periodic with period 1. The initial condition for the phase is defined, as per equation (2.4), for a chosen macroscale saturation, *S*, as $\phi_{0}(x\;x,y\;y,0)=\bar{\phi }(S(x\;x),y\;y)$, where

2.11 $$S=\frac{1}{∥B∥}\int_{B}\phi_{0}\;dy\;y.$$

We are interested in the behaviour of the fluids and exudates on a timescale that is much longer than *τ*_−1_, i.e. longer than the time it takes for the air–water capillary interfaces to equilibrate on the pore scale. Hence, we are only interested in the steady-state behaviour of equations (2.10). However, as equation (2.10c) is nonlinear, the solution to (2.10a) is dependent on the initial condition. Therefore, to find the steady-state solutions of *p*_0_, *μ*_0_, *ϕ*_0_ and *c*_0_ from equations (2.10), it is necessary to include the time derivative in equation (2.10a) and numerically integrate to the steady state.

While we have to solve parts of equations (2.10) numerically, we are able to determine certain properties of the steady-state solution by analysing these equations. These properties will enable us to progress through the homogenization procedure without knowing the precise solution to equations (2.10). The numerical solutions can then be calculated to parametrize the resulting equations. We note that, at steady state, equation (2.10a) is satisfied for any *μ*_0_∼*μ*_0_(***x***), i.e. *μ*_0_ is constant in ***y***, and equation (2.10b) is satisfied for any *p*_0_∼*p*_0_(***x***). Following Daly & Roose [8], we note that at steady state *ϕ*_0_ is a function of ***x*** and ***y*** and can be written as *ϕ*_0_ = *S*(***x***, *τ*_0_, *τ*_1_) + *ϕ*^(*m*)^_0_(*S*(***x***, *τ*_0_, *τ*_1_),***y***, *τ*_−1_), where *ϕ*^(*m*)^_0_(*S*(***x***, *τ*_0_, *τ*_1_),***y***, *τ*_−1_) is the modulated part of *ϕ*_0_ with zero average, and the only ***x*** dependence in *ϕ*_0_ comes through the saturation *S*(***x***). Also, at steady state, equations (2.10d,*g*) have the solution $c_{0}=\bar{C}(x\;x,\tau_{0},\tau_{1})\phi_{0}(x\;x,y\;y,\tau_{-1},\tau_{0},\tau_{1})$, where $\bar{C}$ is the macroscale component of *c*_0_.

As the value of *μ*_0_ is determined by equations (2.10a,*c*,*e*), it depends on the initial condition, $\bar{\phi }$, the surface tension, *γ*(*c*_0_) and the contact angle *θ*(*c*_0_). We assume that, $\gamma (c_{0})=\gamma [\bar{C}(x\;x,\tau_{0},\tau_{1})]$ and $\theta (c_{0})=\theta [\bar{C}(x\;x,\tau_{0},\tau_{1})]$, i.e. the surface tension and contact angle do not depend on higher order terms in *c*. Rather, they are only dependent on the macroscale concentration of exudate and not the position of the air–water interface. As a result, we can scale the surface tension out of equations (2.10), using the scalings ${\bar{\tau }}_{-1}=\tau_{-1}\gamma (\bar{C})$ and ${\bar{\mu }}_{0}=\mu_{0}/\gamma (\bar{C})$, to obtain a set of equations which depend only on the geometry and contact angle,

2.12*a* $$\begin{array}{rl} & \frac{\partial \phi_{0}}{\partial {\bar{\tau }}_{-1}}=\Upsilon {\nabla \;\nabla }_{y}\cdot \frac{M_{0}}{\eta_{0}}{\nabla \;\nabla }_{y}{\bar{\mu }}_{0},\;y\;y\in B, \end{array}$$

2.12*b* $$\begin{array}{rl} & {\bar{\mu }}_{0}=\lambda^{-1}f^{\prime }(\phi_{0})-\lambda {\nabla \;\nabla }_{y}^{2}\phi_{0},\;y\;y\in B, \end{array}$$

2.12*c* $$\begin{array}{rl} & \hat{n\;n}\cdot \frac{M_{0}}{\eta_{0}}{\nabla \;\nabla }_{y}{\bar{\mu }}_{0}=0,\;y\;y\in \partial B, \end{array}$$

2.12*d* $$\begin{array}{rl} & \hat{n\;n}\cdot \nabla \;\nabla \phi_{0}=|{\nabla \;\nabla }_{y}\phi_{0}|\cos(\theta (\bar{C})),\;y\;y\in \partial B \end{array}$$

2.12*e* $$\begin{array}{rl} \;\text{and}\; & \phi_{0}(t=0)=\bar{\phi }. \end{array}$$

The result is that, for fixed values of *S* and *θ*, we can calculate ${\bar{\mu }}_{0}$ by solving equations (2.12). By repeating this for a range of different saturation values while keeping the contact angle fixed, we obtain

2.13 $$F[S,\theta (\bar{C})]=\lim_{{\bar{\tau }}_{-1}\to \infty }\{{\bar{\mu }}_{0}[S,\theta (\bar{C})]\},$$

where $F[S,\theta (\bar{C})]$ is the SWCC for contact angle $\theta (\bar{C})$. Hence, at steady state in *τ*_−1_ we write

2.14 $$\mu_{0}=\gamma (\bar{C})F[S,\theta (\bar{C})].$$

Equation (2.14) is the SWCC, where for constant concentration, $F[S,\theta (\bar{C})]$, is the numerical analogue to fitting the well-known van-Genuchten or Brooks and Corey models to an experimentally measured SWCC [6,7]. In this case, however, the SWCC will be different for different contact angles. Hence, if $\theta (\bar{C})$ is known then the SWCC can be calculated as a function of both saturation and concentration. In reality, it will not be possible to solve equations (2.12) for all values of *S* and $\bar{C}$. However, the curve can be numerically generated by solving for a fixed set of *S* and $\bar{C}$ and using interpolation to obtain the function at all saturation and concentration values.

At this point, we return to the expression for the viscosity, equation (2.7b). In order to proceed, we need to address two issues with this equation. Firstly, since the Cahn–Hilliard equation allows the phase to be of order λ outside the defined range of (0, 1), inputting *ϕ*_0_ directly into the viscosity can result in negative viscosities, which are not physical. Secondly, as equation (2.7b) is not in the form *η*(***x***,***y***) = *η*^*y*^(***y***)*η*^*x*^(***x***) it is impossible to separate the ***x*** and ***y*** dependence. Hence, we approximate the leading-order viscosity as

2.15 $$\eta_{0}=\left[\eta^{\left(a\right)}-(\eta^{\left(w\right)}-\eta^{\left(a\right)})\left(\frac{\phi_{0}-\min(\phi_{0})}{\max(\phi_{0})-\min(\phi_{0})}\right)\right]\eta^{\left(w\right)}(\bar{C}(x))=\eta_{0}^{\phi }\eta^{\left(w\right)}(\bar{C}),$$

where *ϕ*_0_ has been scaled using the maximum and minimum values to force the value to be strictly in the interval (0,1). The effect of having the macroscale viscosity outside the phase terms is that both water and air are dependent on the macroscale concentration, rather than just the water. As *η*^(a)^≪*η*^(w)^, the effect of this approximation should be small. We will quantify the effect of this approximation in §3.

#### (ii) $O(\varepsilon^{0})$

We now collect terms of $O(\varepsilon^{0})$ from equations (2.8). Our aim is to find an approximation for the effect of large-scale pressure and concentration gradients on the local phase, velocity, pressure and concentration. The steps required to derive these equations are included in the electronic supplementary material S1. We are interested in the behaviour of the porous medium on the long timescale, i.e. we assume that the equilibration of the air–water interface occurs on a timescale *τ*_−1_ that is much quicker than the one associated with bulk fluid movement. In addition, we show that the $O(\epsilon^{0})$ fluxes balance and the resulting equations are independent of *τ*_0_, electronic supplementary material S1. Hence, we can write

2.16*a* $$\begin{array}{rl} & u\;u_{0}\cdot {\nabla \;\nabla }_{y}\phi_{0}=\Upsilon \left({\nabla \;\nabla }_{y}\cdot \frac{M_{0}}{\eta_{0}}{\nabla \;\nabla }_{y}\mu_{1}+{\nabla \;\nabla }_{y}\cdot \frac{M_{0}}{\eta_{0}}{\nabla \;\nabla }_{x}\mu_{0}\right),\;y\;y\in B, \end{array}$$

2.16*b* $$\begin{array}{rl} & {\nabla \;\nabla }_{y}\cdot \eta_{0}\sigma \;\sigma_{0y}-{\nabla \;\nabla }_{x}p_{0}-{\nabla \;\nabla }_{y}p_{1}-\phi_{0}{\nabla \;\nabla }_{y}\mu_{1}-\phi_{0}{\nabla \;\nabla }_{x}\mu_{0}=\phi_{0}g{\hat{e\;e}}_{3},\;y\;y\in B, \end{array}$$

2.16*c* $$\begin{array}{rl} & {\nabla \;\nabla }_{y}\cdot u\;u_{0}=0,\;y\;y\in B \end{array}$$

2.16*d* $$\begin{array}{rl} \;\text{and}\; & \mu_{1}=\gamma (\bar{C})\lambda^{-1}f^{\prime \prime }(\phi_{0})\phi_{1}-\lambda {\nabla \;\nabla }_{y}^{2}\phi_{1},\;y\;y\in B, \end{array}$$

where the boundary conditions are

2.16*e* $$u\;u_{0}=0,\;\hat{n\;n}\cdot {\nabla \;\nabla }_{x}\phi_{0}+\hat{n\;n}\cdot {\nabla \;\nabla }_{y}\phi_{1}=|{\nabla \;\nabla }_{y}\phi_{1}+{\nabla \;\nabla }_{x}\phi_{0}|\cos(\theta (\bar{C})),\;y\;y\in \partial B,$$

and

2.16*f* $$\hat{n\;n}\cdot \frac{M_{0}}{\eta_{0}}{\nabla \;\nabla }_{x}\mu_{0}+\hat{n\;n}\cdot \frac{M_{0}}{\eta_{0}}{\nabla \;\nabla }_{y}\mu_{1}=0,\;y\;y\in \partial B,$$

and the exudate transport equation is

2.16*g* $${\nabla \;\nabla }_{y}\cdot (\phi_{0}{\nabla \;\nabla }_{y}c_{1}-c_{1}{\nabla \;\nabla }_{y}\phi_{0})=-{\nabla \;\nabla }_{y}\cdot (\phi_{0}^{2}{\nabla \;\nabla }_{x}\bar{C}),\;y\;y\in B,$$

with boundary condition

2.16*h* $$\hat{n\;n}\cdot (\phi_{0}{\nabla \;\nabla }_{y}c_{1}-c_{1}{\nabla \;\nabla }_{y}\phi_{0})=-\hat{n\;n}\cdot \phi_{0}^{2}{\nabla \;\nabla }_{x}\bar{C},\;y\;y\in \partial B.$$

We note that the advection term from equation (2.8g) is not present as transport is dominated by the diffusion term when λ→0, as shown in electronic supplementary material S1. Equations (2.16) describe the phase, velocity and pressure of the air and water phases and the exudate concentration of the water on the short space scale. We require that these equations are satisfied for all possible combinations of gradients on the large scale. Hence, we look for solutions in a separable form

2.17*a* $$\begin{array}{rl} & u\;u_{0}=\sum_{k}\kappa \;\kappa_{k}^{\mu }(y\;y)\frac{\partial_{x_{k}}\mu_{0}(x\;x)}{\eta [\bar{C}(x\;x)]}+\kappa \;\kappa_{k}^{p}(y\;y)\frac{\partial_{x_{k}}p_{0}(x\;x)}{\eta [\bar{C}(x\;x)]}+\kappa \;\kappa^{g}(y\;y)\frac{g}{\eta [\bar{C}(x\;x)]}, \end{array}$$

2.17*b* $$\begin{array}{rl} & \mu_{1}=\sum_{k}\chi_{k}^{\mu }(y\;y)\partial_{x_{k}}\mu_{0}(x\;x)+\chi_{k}^{p}(y\;y)\partial_{x_{k}}p_{0}(x\;x)+\chi^{g}(y\;y)g, \end{array}$$

2.17*c* $$\begin{array}{rl} & p_{1}=\sum_{k}\omega_{k}^{\mu }(y\;y)\partial_{x_{k}}\mu_{0}(x\;x)+\omega_{k}^{p}(y\;y)\partial_{x_{k}}p_{0}(x\;x)+\omega^{g}(y\;y)g, \end{array}$$

2.17*d* $$\begin{array}{rl} & \phi_{1}=\frac{1}{\gamma [\bar{C}(x\;x)]}\sum_{k}\psi_{k}^{\mu }(y\;y)\partial_{x_{k}}\mu_{0}(x\;x)+\psi_{k}^{p}(y\;y)\partial_{x_{k}}p_{0}(x\;x)+\psi^{g}(y\;y)g \end{array}$$

2.17*e* $$\begin{array}{rl} \;\text{and}\; & c_{1}=\sum_{k}\xi^{c}(y\;y)\partial_{x_{k}}\bar{C}(x\;x). \end{array}$$

We substitute the solutions in a separable form, equations (2.17), into equations (2.16) and gather terms dependent on *μ*_0_, the macroscale capillary pressure, to get,

2.18*a* $$\begin{array}{rl} & \kappa \;\kappa_{k}^{\mu }\cdot {\nabla \;\nabla }_{y}\phi_{0}=\Upsilon \left({\nabla \;\nabla }_{y}\cdot \frac{M_{0}}{\eta_{0}^{\phi }}{\nabla \;\nabla }_{y}\chi_{k}^{\mu }+{\nabla \;\nabla }_{y}\cdot \frac{M_{0}}{\eta_{0}^{\phi }}{\hat{e\;e}}_{k}\right),\;y\;y\in B, \end{array}$$

2.18*b* $$\begin{array}{rl} & {\nabla \;\nabla }_{y}\cdot \eta_{0}^{\phi }\sigma \;\sigma_{0y}^{\mu }-{\nabla \;\nabla }_{y}\omega_{k}^{\mu }-\phi_{0}{\nabla \;\nabla }_{y}\chi_{k}^{\mu }-\phi_{0}{\hat{e\;e}}_{k}=0,\;y\;y\in B, \end{array}$$

2.18*c* $$\begin{array}{rl} & {\nabla \;\nabla }_{y}\cdot \kappa \;\kappa_{k}^{\mu }=0,\;y\;y\in B \end{array}$$

2.18*d* $$\begin{array}{rl} \;\text{and}\; & \chi_{k}^{\mu }=\lambda^{-1}f^{\prime \prime }(\phi_{0})\psi_{k}^{\mu }-\lambda {\nabla \;\nabla }_{y}^{2}\psi_{k}^{\mu },\;y\;y\in B, \end{array}$$

where ${\sigma \;\sigma }_{0y}^{\mu }\;=\;{\nabla \;\nabla }_{y}{\kappa \;\kappa }_{k}^{\mu }\;+\;{\left({\nabla \;\nabla }_{y}{\kappa \;\kappa }_{k}^{\mu }\right)}^{T}$. The corresponding boundary conditions are

2.18*e* $$\kappa \;\kappa_{k}^{\mu }=0,\;\hat{n\;n}\cdot {\nabla \;\nabla }_{y}\psi_{k}^{\mu }=|{\nabla \;\nabla }_{y}\psi_{k}^{\mu }|\cos(\theta (\bar{C})),\;y\;y\in \partial B$$

and

2.18*f* $$\hat{n\;n}\cdot M_{0}{\hat{e\;e}}_{k}+\hat{n\;n}\cdot M_{0}{\nabla \;\nabla }_{y}\chi_{k}^{\mu }=0,\;y\;y\in \partial B.$$

Equations (2.18) determine the fluid velocity driven by a large-scale variation in capillary pressure. Physically, this corresponds to the difference in pressure between the two phases. Hence, in the limit λ → 0, only the water phase is directly driven by the capillary pressure. The air phase is not directly driven by the capillary pressure, but can be set in motion by the water velocity at the air–water boundary.

Next, we gather terms dependent on the macroscale combined pressure, *p*_0_,

2.19*a* $$\begin{array}{rl} & \kappa \;\kappa_{k}^{p}\cdot {\nabla \;\nabla }_{y}\phi_{0}=\Upsilon \left({\nabla \;\nabla }_{y}\cdot \frac{M_{0}}{\eta_{0}^{\phi }}{\nabla \;\nabla }_{y}\chi_{k}^{p}\right),\;y\;y\in B, \end{array}$$

2.19*b* $$\begin{array}{rl} & {\nabla \;\nabla }_{y}\cdot \eta_{0}^{\phi }\sigma \;\sigma_{0y}^{p}-{\hat{e\;e}}_{k}-{\nabla \;\nabla }_{y}\omega_{k}^{p}-\phi_{0}{\nabla \;\nabla }_{y}\chi_{k}^{p}=0,\;y\;y\in B, \end{array}$$

2.19*c* $$\begin{array}{rl} & {\nabla \;\nabla }_{y}\cdot \kappa \;\kappa_{k}^{p}=0,\;y\;y\in B \end{array}$$

2.19*d* $$\begin{array}{rl} \;\text{and}\; & \chi_{k}^{p}=\lambda^{-1}f^{\prime \prime }(\phi_{0})\psi_{k}^{p}-\lambda {\nabla \;\nabla }_{y}^{2}\psi_{k}^{p},\;y\;y\in B, \end{array}$$

where ${\sigma \;\sigma }_{0y}^{p}\;=\;{\nabla \;\nabla }_{y}{\kappa \;\kappa }_{k}^{p}\;+\;{\left({\nabla \;\nabla }_{y}\;{\kappa \;\kappa }_{k}^{p}\right)}^{T}$. The corresponding boundary conditions are

2.19*e* $$\kappa \;\kappa_{k}^{p}=0,\;\hat{n\;n}\cdot {\nabla \;\nabla }_{y}\psi_{k}^{p}=|{\nabla \;\nabla }_{y}\psi_{k}^{p}|\cos(\theta (\bar{C})),\;y\;y\in \partial B$$

and

2.19*f* $$\hat{n\;n}\cdot M_{0}{\nabla \;\nabla }_{y}\chi_{k}^{p}=0,\;y\;y\in \partial B.$$

Equations (2.19) determine the fluid velocity due to a unit pressure gradient, in this case both the air and water phases are driven by the combined pressure, *p*_0_.

Next, we gather terms dependent on gravity, *g*,

2.20*a* $$\begin{array}{rl} & \kappa \;\kappa^{g}\cdot {\nabla \;\nabla }_{y}\phi_{0}=\Upsilon \left({\nabla \;\nabla }_{y}\cdot \frac{M_{0}}{\eta_{0}^{\phi }}{\nabla \;\nabla }_{y}\chi^{g}\right),\;y\;y\in B, \end{array}$$

2.20*b* $$\begin{array}{rl} & {\nabla \;\nabla }_{y}\cdot \eta_{0}^{\phi }\sigma \;\sigma_{0y}^{g}-{\nabla \;\nabla }_{y}\omega^{g}-\phi_{0}{\nabla \;\nabla }_{y}\chi^{g}=\phi_{0}{\hat{e\;e}}_{k},\;y\;y\in B, \end{array}$$

2.20*c* $$\begin{array}{rl} & {\nabla \;\nabla }_{y}\cdot \kappa \;\kappa^{g}=0,\;y\;y\in B \end{array}$$

2.20*d* $$\begin{array}{rl} \;\text{and}\; & \chi^{g}=\lambda^{-1}f^{\prime \prime }(\phi_{0})\psi^{g}-\lambda {\nabla \;\nabla }_{y}^{2}\psi^{g},\;y\;y\in B, \end{array}$$

where ${\sigma \;\sigma }_{0y}^{g}\;=\;{\nabla \;\nabla }_{y}{\kappa \;\kappa }^{g}\;+\;{\left({\nabla \;\nabla }_{y}\;{\kappa \;\kappa }^{g}\right)}^{T}$. The corresponding boundary conditions are

2.20*e* $$\kappa \;\kappa^{g}=0,\;\hat{n\;n}\cdot {\nabla \;\nabla }_{y}\psi^{g}=|{\nabla \;\nabla }_{y}\psi^{g}|\cos(\theta (\bar{C})),\;y\;y\in \partial B$$

and

2.20*f* $$\hat{n\;n}\cdot M_{0}{\nabla \;\nabla }_{y}\chi^{g}=0,\;y\;y\in \partial B.$$

Equations (2.20) determine the fluid velocity due to gravity. As we chose to neglect the effect of gravity on the much less dense air phase, only the water phase is directly driven by gravity. Any induced movement of the air phase comes from the effect of water movement at the air–water interface.

Finally, we gather terms dependent on *c*_0_,

2.21*a* $${\nabla \;\nabla }_{y}\cdot (\phi_{0}{\nabla \;\nabla }_{y}\xi_{k}^{c}-\xi_{k}^{c}{\nabla \;\nabla }_{y}\phi_{0})=-{\nabla \;\nabla }_{y}\cdot (\phi_{0}^{2}{\hat{e\;e}}_{k}),\;y\;y\in B,$$

with boundary condition

2.21*b* $$\hat{n\;n}\cdot (\phi_{0}{\nabla \;\nabla }_{y}\xi_{k}^{c}-\xi_{k}^{c}\nabla_{y}\phi_{0})=-\hat{n\;n}\cdot \phi_{0}^{2}{\hat{e\;e}}_{k},\;y\;y\in \partial B.$$

Equations (2.21) determine the local geometry-dependent diffusion offered by the soil pore structure and the position of the air–water interface. Physically, this will be combined with the unimpeded diffusion coefficient to calculate the effective diffusion coefficient in the water phase as a function of saturation.

Cell problems (2.18)–(2.21), for known *ϕ*_0_ and $\theta (\bar{C})$, provide a complete description of how the pore scale geometry and physical processes are dependent on large-scale variations in capillary pressure, combined pressure, acceleration due to gravity and the concentration of root exudates. In the next section, we will derive equations for terms of order *ε*^1^, which will relate these pore scale behaviours to the macroscale flow.

#### (iii) $O(\varepsilon^{1})$

The final step in the homogenization procedure is to expand equations (2.8) to $O(\varepsilon^{1})$. Then, by applying a solvability condition and taking a volume average over the domain *B*, we find a series of macroscale equations that describe the averaged movement of air, water and exudate through the soil. Finally, we take the limit λ → 0 to obtain

2.22*a* $$\begin{array}{rl} & ∥B∥\frac{\partial S}{\partial \tau_{1}}+{\nabla \;\nabla }_{x}\cdot U\;U=0,\;x\;x\in \Omega , \end{array}$$

2.22*b* $$\begin{array}{rl} & {\nabla \;\nabla }_{x}\cdot \bar{U\;U}=0,\;x\;x\in \Omega \end{array}$$

2.22*c* $$\begin{array}{rl} \;\text{and}\; & ∥B∥\left(S\frac{\partial \bar{C}}{\partial \tau_{1}}+\bar{C}\frac{\partial S}{\partial \tau_{1}}\right)+{\nabla \;\nabla }_{x}\cdot (\bar{C}U\;U)-{\nabla \;\nabla }_{x}\cdot D(∥B∥SI\;I+D_{\mathrm{eff}}){\nabla \;\nabla }_{x}\bar{C}=0,\;x\;x\in \Omega , \end{array}$$

where

2.22*d* $$U\;U=\frac{K[S,\theta (\bar{C})]}{\eta^{\left(w\right)}(\bar{C})}{\nabla \;\nabla }_{x}\mu_{0}+\frac{b[S,\theta (\bar{C})]}{\eta^{\left(w\right)}(\bar{C})}{\nabla \;\nabla }_{x}p_{0}+\frac{b_{g}[S,\theta (\bar{C})]}{\eta^{\left(w\right)}(\bar{C})}{\hat{e\;e}}_{3}g$$

and

2.22*e* $$\bar{U\;U}=\frac{\bar{K}[S,\theta (\bar{C})]}{\eta^{\left(w\right)}(\bar{C})}{\nabla \;\nabla }_{x}\mu_{0}+\frac{\bar{b}[S,\theta (\bar{C})]}{\eta^{\left(w\right)}(\bar{C})}{\nabla \;\nabla }_{x}p_{0}+\frac{{\bar{b}}_{g}[S,\theta (\bar{C})]}{\eta^{\left(w\right)}(\bar{C})}{\hat{e\;e}}_{3}g,$$

and we recall the SWCC is given by

2.22*f* $$\mu_{0}=F\left[S,\theta (\bar{C})\right]\gamma (\bar{C}).$$

The derivation of these equations involves a large number of steps and the details are included in the electronic supplementary material S1. Equation (2.22a) is equivalent to the macroscale equation from Daly & Roose [8] for $\eta^{\left(w\right)}(\bar{C})=\gamma (\bar{C})=1$ and $\theta (\bar{C})=\theta (0)$, i.e. where the viscosity and surface tension are constant and not dependent on the root exudate concentration. Note that if it is also assumed that the pressure in the air phase is constant, as used in deriving Richards' equation, equation (2.22a) reduces to Richards' equations. If gravity is also neglected, then Buckingham–Darcy's Law is also recovered. Equation (2.22a) describes how the macroscale saturation varies with time depending on the concentration-dependent viscosity and surface tension and it is related to the pore scale behaviour by the parameters $K[S,\theta (\bar{C})]$, $b[S,\theta (\bar{C})]$ and $b_{g}[S,\theta (\bar{C})]$ that are defined using volume averages

2.23*a* $$\begin{array}{rl} K[S,\theta (\bar{C})] & =\int_{B}\phi_{0}\kappa \;\kappa_{k}^{\mu }⊗{\hat{e\;e}}_{k}\;dy\;y, \end{array}$$

2.23*b* $$\begin{array}{rl} b[S,\theta (\bar{C})] & =\int_{B}\phi_{0}\kappa \;\kappa_{k}^{p}⊗{\hat{e\;e}}_{k}\;dy\;y \end{array}$$

2.23*c* $$\begin{array}{rl} \;\text{and}\;b_{g}[S,\theta (\bar{C})] & =\int_{B}\phi_{0}\kappa \;\kappa^{g}⊗{\hat{e\;e}}_{3}\;dy\;y. \end{array}$$

Equations (2.23a–*c*) describe the velocity of the water phase averaged over the pore scale domain driven by capillary pressure, combined pressure and gravity, respectively. Equation (2.22b) ensures the conservation of mass for the saturation equation (2.22a), where the concentration-dependent viscosity and surface tension are present, and are related to the pore scale behaviour by,

2.24*a* $$\begin{array}{rl} \bar{K}[S,\theta (\bar{C})] & =\int_{B}\kappa \;\kappa_{k}^{\mu }⊗{\hat{e\;e}}_{k}\;dy\;y, \end{array}$$

2.24*b* $$\begin{array}{rl} \bar{b}[S,\theta (\bar{C})] & =\int_{B}\kappa \;\kappa_{k}^{p}⊗{\hat{e\;e}}_{k}\;dy\;y \end{array}$$

2.24*c* $$\begin{array}{rl} \;\text{and}\;{\bar{b}}_{g}[S,\theta (\bar{C})] & =\int_{B}\kappa \;\kappa^{g}⊗{\hat{e\;e}}_{3}\;dy\;y. \end{array}$$

Equations (2.24a–*c*) describe the velocity of both the air and water phases averaged over the pore scale domain driven by capillary pressure, combined pressure and gravity, respectively. Finally, equation (2.22c) describes the movement of exudate on the macroscale, driven by diffusion and fluid movement, and is related to the pore scale behaviour by,

2.25 $$D_{\mathrm{eff}}[S,\theta (\bar{C})]=\int_{B}(\phi_{0}{\nabla \;\nabla }_{y}\xi_{k}^{c}-\xi_{k}^{c}{\nabla \;\nabla }_{y}\phi_{0})⊗{\hat{e\;e}}_{k}\;dy\;y.$$

Equation (2.25) is the average geometry-dependent diffusion on the pore scale domain due to the soil pore structure and position of the air–water interface. Equations (2.22) provide a complete description of how saturation, pressure and concentration change over time. The equations are coupled through the viscosity, surface tension, saturation-dependent diffusion constant and the SWCC which is calculated numerically from equation (2.12). In addition, the soil geometry is captured through the parameters given in equations (2.23)–(2.25), which are based on the cell problems (2.18)–(2.21).

This is an advancement from the work of Daly & Roose [8] as we have combined the equations for fluid flow with the equations for exudate diffusion. If we neglect diffusion and root exudates then equations (2.22) reduce to those derived by Daly & Roose [8]. Alternatively, if we wanted to consider diffusion of solutes, which did not directly influence the properties of water, then setting $\gamma (\bar{C})=\eta^{\left(w\right)}(\bar{C})=1$ and $\theta (\bar{C})=\theta (0)$ would provide a partially coupled set of equations describing the movement of air and water, from which the diffusion of solutes, which do not bind to the soil particle surfaces, could be calculated.

## 3. Validation of homogenization procedure

The homogenization procedure involved a large number of mathematical steps and assumptions. In order to test the accuracy of these assumptions and to transparently demonstrate the application of the method, we show that the macroscale model, equations (2.22) presented above, provides an accurate approximation of the pore scale equations, (2.8). We do this using an idealized geometry for two cases; firstly, we compare the homogenized equations to the pore scale equations with the original viscosity, equation (2.7b). Secondly, we compare the homogenized model to the original equations with the assumed viscosity, equation (2.15). Our overall aim is to test the accuracy of the mathematical steps, not to compare the predictions of this model with experimental results, which is beyond the scope of this paper.

### (a) Test geometry

We consider a soil column in which the saturation is perturbed by subtracting a linear function from the initial phase configuration. This drives a re-equilibration of both the fluid phase and the local concentration of exudate. All equations are solved numerically using Comsol Multiphysics (v5.2a). In order to simplify the analysis, we consider the case $\bar{U\;U}=0$, corresponding to the case in which the air viscosity is much smaller than the water viscosity. In this case, we also assume *p*_0_ is constant and the effects of gravity can be neglected.

For the purposes of validation, we focus on the effect of viscosity on water movement and exudate diffusion. Hence, we choose $\gamma (\bar{C})=1$ and note that, in the case $\bar{U\;U}=0$, the only effect of the surface tension changing is a variation in the timescales which appear in equation (2.22). We also make the simplification $\theta (\bar{C})=0$, corresponding to a fully wetted soil. Mathematically, the contact angle features in all the cell problems either directly, or indirectly through the function *ϕ*_0_. The effect of changing the contact angle in this way has been investigated by Daly *et al.* [13]. Hence, here we neglect these changes and focus only on viscosity.

**Figure 2.**
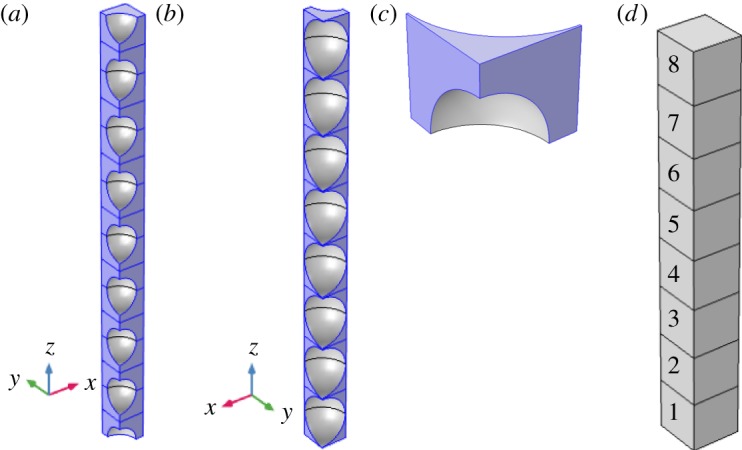
Geometry used for validation. In (*a*–*c*), the blue areas show the pore space and grey shows soil particles boundaries. (*a*) Idealized pore space geometry for test case. The soil particles shown have radius 0.35 [dimensionless]; (*b*) idealized pore space geometry for test case. The soil particles shown have radius 0.49 [dimensionless]; (*c*) pore geometry used for cell problem; (*d*) geometry used for homogenized equation, each cube is 1 × 1 × 1 [dimensionless]. The numbers indicate the unit cell labels. (Online version in colour.)

We consider the geometry shown in figure 2*a*,*b*. This idealized soil physical structure is composed of soil particles of two different sizes. The soil geometry is built from repetition of a unit cell. At the centre of the cell, we have placed a spherical soil particle of radius 0.49 [dimensionless] and at the corners of the cell we have placed a spherical soil particle of radius 0.35 [dimensionless]. These two sizes have been chosen to force the geometry to exhibit wetting–drying hysteresis, i.e. the SWCC will exhibit different values depending on whether the water content is increasing or decreasing. This hysteresis requires pores of different sizes, something which does not occur for a single soil particle size for a perfectly packed structure. The full geometry is formed from eight repetitions of the unit cell (which we label unit 1 to unit 8). However, to reduce the computational load we have used symmetry to quarter the domain, see figure 2*b*. The cell problem is calculated on one-eighth of the unit cell and it is of size 0.5^3^ (dimensionless) (figure 2*c*). It was meshed with tetrahedral elements, maximum size 0.0335 and minimum size 0.002, and one boundary layer on the surfaces of the soil particles. This mesh was sufficient to resolve the interface with width 0.02 (dimensionless) using quadratic elements. A mesh refinement study was conducted to ensure this was a suitable mesh. The same mesh was used for the full model. The homogenized model used a one-dimensional mesh with 96 evenly spaced elements. All the models were solved using the MUMPS solver with Newton's method, with a constant damping of 1 so that it has quadratic convergence, within the fully coupled solver in Comsol Multiphysics. The solution was declared converged when an absolute tolerance of 10^−4^ was reached.

The homogenized equations (2.22), are applied to a unit cuboid (figure 2*d*). In order to ensure that the homogenized equations (2.22) converge, we require that there are enough repetitions of the unit cell such that *L*_*y*_/*L*_*x*_ = *ε*≪1. As the full equations are computationally expensive, we also require that we have a small enough domain that the resulting equations are computationally tractable. By gradually increasing the number of repetitions of the unit cell, we found that eight repetitions was sufficient for the homogenized equations (2.22) to converge. However, good convergence between the models can also be found for as few as four repetitions.

### (b) Numerical results

**Figure 3.**
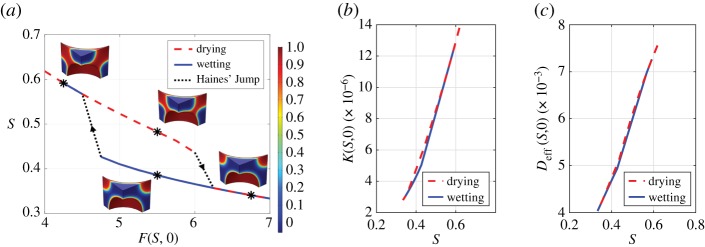
Solutions calculated on the cell geometry (figure 2*c*), using the cell problems (2.12), (2.18) and (2.21). *K*(*S*, 0) is the saturation-dependent hydraulic conductivity for a 0° contact angle and *D*_eff_(*S*, 0) is the saturation-dependent geometry-dependent diffusion for a 0° contact angle. (*a*) Soil–water characteristic curve calculated using equations (2.12). Four different phase topologies are shown with asterisks that indicate the corresponding points on the graph. The colour bar represents the value of *ϕ*_0_, i.e. 1 is water, 0 is air. The arrowheads indicate the transition between states. (*b*) Hydraulic conductivity calculated using equations (2.18). (*c*) Geometry-dependent diffusion calculated using equations (2.21). (Online version in colour.)

Before we solved the homogenized equations, the cell problem, equations (2.12), (2.18) and (2.21), were evaluated for a range of dimensionless capillary pressures between −7 and −4 [dimensionless] with λ = 2 × 10^−3^ [dimensionless] and *Υ* = 1 [dimensionless]. As we have neglected the effects of combined pressure and gravity, it is not necessary to solve cell problems (2.19) and (2.20). We solved equations (2.12), (2.18) and (2.21) for both increasing and decreasing capillary pressures in order to evaluate the wetting and drying curves (figure 3*a*). The parameters *F*(*S*, 0) and, hence, the effective parameters $K[S,\theta (\bar{C})]$ and *D*_eff_(*S*) exhibit wetting drying hysteresis and Haines' jumps [32], where the topology of the air–water interfaces changes resulting in a large change in saturation for a small change in capillary pressure. These parameters feed into equations (2.22) and are used to calculate the solution to the homogenized equations.

To establish the initial configuration for the phase of the full model, equation (2.8), the dimensionless capillary pressure was set to −4.25 and the model was run to steady state, resulting in an average saturation of 0.6 over each unit in the column. This was outside the hysteresis loop of the SWCC at the wet end (figure 3*a*). The saturation was perturbed in the full model by subtracting a linear function from the initial configuration of the phase, i.e. ${\bar{\phi }}_{0}={\overset{ˇ}{\phi }}_{0}-A(1-(h/8))$, where ${\overset{ˇ}{\phi }}_{0}$ is the unperturbed phase field, *A* is the magnitude of the perturbation and *h* is the vertical position of the column. For *A* > 0, this resulted in a greater saturation at the top of the column and lower saturation at the bottom. The equivalent condition was applied to the homogenized model using the expression *S*(*t* = 0) = 0.6 − *A*(1 − (*h*/8)). Two perturbations were used, a small perturbation with *A* = 0.1 [dimensionless] and a large perturbation with *A* = 0.25 [dimensionless] that allows for comparison between the models where Haines' jumps and hysteresis are present. The initial concentration of the root exudate was set to 0.2 [dimensionless] for the small perturbation and 0.5 [dimensionless] for the large perturbation. The expression for the concentration-dependent viscosity was chosen to be

3.1 $$\eta^{\left(w\right)}(\bar{C})=(q{\bar{C}}^{2}+1)\times (\bar{C}>0),$$

where *q* is 1059. This value comes from fitting the function to the concentration versus viscosity relation measured for barley root exudate from Naveed *et al.* [2] (M Naveed 2017, unpublished data, see electronic supplementary material, figure S1.1).

**Figure 4.**
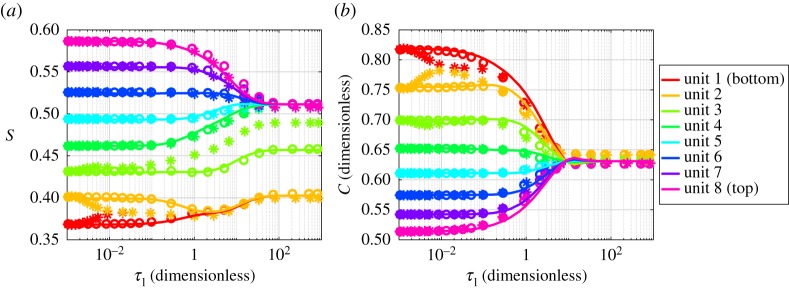
Comparison of full model with original viscosity (*), full model with assumed viscosity (o) and homogenized model (solid line) for the large perturbation. The colours represent the different geometry units in the soil column. *S* is the saturation, $\bar{C}$ is the macroscale exudate concentration and *τ*_1_ is the slow time scale. If time is dimensionalized using the values $\bar{\gamma }=0.072\;N\;m^{-1}$, ${\tilde{L}}_{x}=1\;m$ and ${\tilde{\eta }}^{\left(w\right)}=8.9\times 10^{-4}\;\mathrm{Pa}\cdot s$, then the time shown on the *x*-axis ranges from 1.46 × 10^−6^ s to 1.46 s. (*a*) Saturation and (*b*) concentration. (Online version in colour.)

We compare the results from the full model and the homogenized model as a function of time. All equations were integrated to steady state and the dynamics can be seen in figure 4. For the large perturbation, evidence of a Haines' jump can be seen in figure 4*a* as not all the unit saturations converge to the same steady-state value. For the full model with assumed viscosity, equation (2.15), units 1 and 2 converge to a saturation of approximately 0.4, unit 3 converges to approximately 0.46, whereas the other units converge to approximately 0.51. The homogenized model exhibits the same behaviour as the full model with the assumed viscosity, and captures the effect of the Haines' jump.

**Figure 5.**
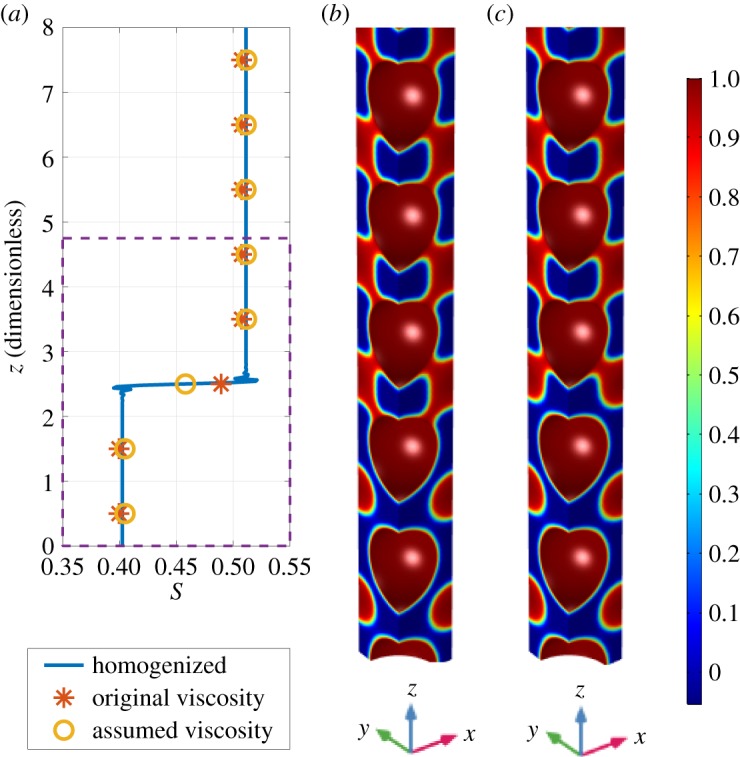
Saturation calculated using homogenized equation along the height of the soil column. The dashed box corresponds to the section of the full models shown in figure 5*b*,*c*. The colour bar shows the values of the phase, *ϕ*, in the full model images; 1 (red) indicates water—water containing root exudate, 0 (blue) indicates air—air without root exudate. The oscillations that can be seen either side of the jump in saturation are due to the choice of numerical solver. (*a*) Steady-state saturation, (*b*) original viscosity and (*c*) assumed viscosity. (Online version in colour.)

The initial exudate concentration also exhibits dynamics, due to the saturation perturbation (figure 4*b*). It can be seen that unit 8, which has the greatest initial saturation, has the smallest exudate concentration due to the dilution effect. The opposite can be observed for unit 1. The homogenized model exhibits behaviour at time 10^1^ [dimensionless], where the exudate concentration of each unit overshoots the steady-state value of 0.63 for the exudate concentration, before all the units converge to 0.63 concentration. For the full model in which the assumed viscosity (2.15) is used, the concentration tends to the same value as the homogenized model, with values of 0.62–0.64 at steady state. The full model in which the original viscosity (2.7b) is used, displays different behaviour to the homogenized model for low saturation, units 1, 2 and 3. At time 10^−2^ [dimensionless], units 1 and 2 in the original viscosity model have converged to the same saturation, 0.38. They also converge to the same saturation at steady state as the homogenized and assumed viscosity model. Unit 3, however, converges to 0.49 at steady state, compared to the saturation of 0.46 reached by the homogenized model. This is due to a topological difference in the final phase configuration as can be seen by comparing figure 5*b*,*c*.

In figure 5, the result of the homogenized model is compared to sections from both of the full models. It can be seen that where the homogenized model has a smaller saturation, caused by the hysteresis and Haines' jumps present in the cell problem results, there is a different phase configuration in the full model. This configuration is different in the original viscosity and assumed viscosity models. The relative differences between the three models are calculated by

3.2 $$E_{r}=2\sqrt{\frac{\sum_{n=1}^{N}{\left(x_{n}^{h}-x_{n}^{f}\right)}^{2}}{\sum_{n=1}^{N}{\left(x_{n}^{h}+x_{n}^{f}\right)}^{2}}}\times 100,$$

where *N* is the total number of time steps taken by the solver for the homogenized model, *x*^h^_*n*_ is the result of the homogenized model at time step *n* and *x*^f^_*n*_ is the result of the full model (either original or assumed viscosity model) interpolated onto the time steps taken by the homogenized model solver. The relative differences are shown in table 1. The maximum errors between individual points of the models are calculated by

3.3 $$E_{m}=2\sqrt{\text{max}\left[\frac{{\left(x_{n}^{h}-x_{n}^{f}\right)}^{2}}{{\left(x_{n}^{h}+x_{n}^{f}\right)}^{2}}\right]}\times 100$$

and are shown in table 2. These values are small (less than or equal to 7%) and similar to the variability between different experimental methods [33]. For the small perturbation all the models exhibit the same behaviour, resulting in a smaller maximum errors (less than or equal to 1.1%) and the results can be seen in the electronic supplementary material, figure S1.2.

**Table 1.** Relative error, *E*_*r*_.

	original viscosity (%)		assumed viscosity (%)	
	saturation	concentration	saturation	concentration
homogenized model	1.82	1.14	0.48	0.95
original viscosity	—	—	0.48	0.65

**Table 2.** Maximum error, *E*_*m*_.

	original viscosity (%)		assumed viscosity (%)	
	saturation	concentration	saturation	concentration
homogenized model	6.85	3.51	2.14	3.03
original viscosity	—	—	6.32	3.68

### (c) Discussion

The homogenized equations provide a good estimate for the saturation and concentration when compared with the full equations. This is particularly true when considering the assumed viscosity model (2.15). Overall, it is not surprising that the homogenized equations provide a better approximation to the assumed viscosity, rather than the original viscosity equations, as the assumed viscosity is the one used in the homogenization scheme. The key difference between these two viscosities is the location at which the Haines' jump is seen to occur.

A significant advantage to the homogenized model is that it is less computationally expensive than the full model. The cell problems for the homogenized model took 20 min and 3 GB ram for each capillary pressure evaluated, in this case 24. This resulted in a total computation time of 8 h on a laptop. The resulting homogenized model took just 2.5 min and 5.8 GB of ram on a standard desktop computer. The full model took up to 26 h and 35 GB ram using a segregated solver on the Iridis 4 supercomputer at the University of Southampton.

The homogenized model captures the fluid behaviour near a Haines jump with maximum error less than 7%. We only consider eight repetitions of the unit cell, which corresponds to *ε* = *L*_*y*_/*L*_*x*_ = 0.125. Formally, we would expect an error of size *ε* to be present in the model. With this in mind, we see that the model provides a much more accurate description of the saturation and concentration than would be expected, i.e. the error is less than 12%. It is interesting to note that the homogenized model is able to capture the hysteresis and Haines jumps despite these violating one of the key assumptions used in the derivation. Formally, we require that *S* varies only on the long spatial scale *L*_*x*_. However, by definition ${\nabla \;\nabla }_{xS}$ is effectively infinite at the Haines jump. In order to analyse the behaviour of the model at this point, future research could use matched asymptotics to attempt to capture the behaviour around a Haines' jump. This would allow the homogenized solution to be defined by appropriate jump conditions.

In §2c, it is assumed that the surface tension and wetting angle are functions of the spatially averaged concentration in order to be able to separate the two length scales of the problem. This assumption is not valid in the case where significant concentrations of the root exudate are absorbed on the air–water interface or soil particle surfaces, and therefore some important physical processes may be ignored. However, in the present model, the concentration is dominated by the macroscale part of the concentration, *C*_0_(*x*), and therefore we would expect that the effects of these missing physics would be small compared with the effect of the spatially averaged concentration. Furthermore, in soil systems, adsorption of organic compounds to mineral surfaces is complex and the physical characteristics of root exudates have only recently been explored [2,14]. Greater experimental characterization would be required before accounting for particle adsorption and air–water interface impacts from surfactants in root exudates, but the model could be adapted to account for these processes in the future.

## 4. Conclusion

In this paper, the Cahn–Hilliard–Stokes equations were combined with a phase-dependent diffusion equation for the root exudates and this full set of equations was homogenized. The homogenized equations were shown to reduce to a exudate concentration-dependent Richards' equation, (2.22a), and a saturation-dependent exudate diffusion equation, (2.22c). This homogenization procedure relied on two key assumptions. Firstly, the fluid–fluid drag coefficient is linearly dependent on the viscosity; and secondly, the viscosity of the air and water are sufficiently different that the error induced by assuming the air viscosity was dependent on exudate concentration was negligible.

In §3, the method was validated by comparing the homogenized, equations (2.22), and assumed viscosity model, equations (2.8) with (2.15), to the original viscosity model, equations (2.8) with (2.7b). The relative errors over the whole simulation for the homogenized and assumed viscosity solutions compared to the original viscosity solution were less than 1%. The maximum errors for a particular time point were less than 7%. This shows that the homogenized model is an appropriate approximation to the full set of equations.

The homogenized model is a much more attractive option for calculating the local macroscale flow and diffusion properties of soil than the pore scale description. This is particularly evident if the pore scale geometry is taken from three-dimensional images, a technique that is becoming more frequently used [13,34]. The model derived in this paper captures the effects of root exudates and could therefore be applied to rhizosphere soil. The homogenized model can also be used to investigate the impact of altered hydraulic properties on the movement of water at large scales and be used to improve our understanding of these effects. Detailed models of this kind have the distinct advantage that they can be used to relate specific porescale soil geometries to the observed macroscopic soil properties. The simulations presented here conserved the root exudate within the domain during wetting and drying. In a natural soil system, root exudates may leach and their physical properties may alter due to microbial decomposition over time [2]. Moreover, adsorption of the exudates onto soil minerals will also affect behaviour, potentially altering properties such as the contact angle and surface tension [2,14]. Future modelling could incorporate these impacts to assess the time variability and longevity of root exudate impacts on flow and retention in soil. This would be particularly useful in exploring how properties change with age along a growing root. Hence, this method could be used to gain greater understanding of soil hydraulic properties in and around the rhizosphere and even lead to the possibility of theoretically simulating the quantity of root exudates required to improve the chances of a plant thriving in particular soil environments. Ultimately this reveals the theoretical potential for plant breeders to manipulate root exudation in the development of crops with more effective root systems. The theory can be further extended to other applications such as geological waste disposal, oil production or oil-spill clean-up problems.

## Supplementary Material

Homogenisation of two fluid flow dependent on exudate concentration.

## Data accessibility

Data supporting this study are available on request from the University of Southampton repository at https://doi.org/10.5258/SOTON/D0609 [35].
