# Supplementary material for: The effect of root exudates on rhizosphere water dynamics
Source: Proc Math Phys Eng Sci. 2018 Sep 5;474(2217):20180149. doi: 10.1098/rspa.2018.0149 (PMC6189581; doi:10.1098/rspa.2018.0149)
Supplement: Homogenisation of two fluid flow dependent on exudate concentration. [file rspa20180149supp1.pdf]

S1. Homogenisation

In this supplementary section we present the details of second and third parts of the homogenisation scheme used for equations (2.8) from the main text. We start at $\mathcal{O}(\varepsilon^0)$.

(a) $\mathcal{O}(\varepsilon^0)$

We collect terms of order ε^0 in equations (2.8) using the expansions (2.9). Using $\boldsymbol{\nabla}_y \mu_0 = 0$, the equations are,

$$\frac{\partial \phi_0}{\partial \tau_0} + \frac{\partial \phi_1}{\partial \tau_{-1}} + \boldsymbol{u}_0 \cdot \boldsymbol{\nabla}_y \phi_0 = \Upsilon \left(\boldsymbol{\nabla}_y \cdot \frac{M_0}{\eta_0} \boldsymbol{\nabla}_y \mu_1 + \boldsymbol{\nabla}_y \cdot \frac{M_0}{\eta_0} \boldsymbol{\nabla}_x \mu_0 \right), \qquad \boldsymbol{y} \in B, \qquad \text{(S1.1}a\text{)}$$

$$\boldsymbol{\nabla}_y \cdot \eta_0 \boldsymbol{\sigma}_{0y} - \boldsymbol{\nabla}_x p_0 - \boldsymbol{\nabla}_y p_1 - \phi_0 \boldsymbol{\nabla}_y \mu_1 + \phi_0 \boldsymbol{\nabla}_x \mu_0 = \phi_0 g \hat{\boldsymbol{e}}_3, \qquad \boldsymbol{y} \in B, \qquad \text{(S1.1}b\text{)}$$

$$\boldsymbol{\nabla}_y \cdot \boldsymbol{u}_0 = 0, \qquad \boldsymbol{y} \in B, \qquad \text{(S1.1}c\text{)}$$

$$\mu_1 = \gamma\left(\bar{C}\right) \left(\lambda^{-1} f''(\phi_0)\phi_1 - \lambda \left(\boldsymbol{\nabla}_y^2 \phi_1 + \boldsymbol{\nabla}_y \cdot \boldsymbol{\nabla}_x \phi_0 + \boldsymbol{\nabla}_x \cdot \boldsymbol{\nabla}_y \phi_0 \right) \right), \qquad \boldsymbol{y} \in B. \qquad \text{(S1.1}d\text{)}$$

Here, we note that we can simplify equation (S1.1d) by noting that,

$$\lambda \boldsymbol{\nabla}_y \cdot \boldsymbol{\nabla}_x \phi_0 = \lambda \boldsymbol{\nabla}_y \cdot \boldsymbol{\nabla}_x \left(S(\boldsymbol{x}) + \phi_0^{(m)}(S(\boldsymbol{x}, \boldsymbol{y})) \right) \qquad \text{(S1.2}a\text{)}$$

$$= \lambda \boldsymbol{\nabla}_y \cdot \frac{\delta \phi_0^{(m)}}{\delta S} \boldsymbol{\nabla}_x S(\boldsymbol{x}) \lesssim \mathcal{O}(1). \qquad \text{(S1.2}b\text{)}$$

This term is one order of λ smaller than all others in equation (S1.1d), and in the limit $\lambda \to 0$ may be neglected from the analysis. The other terms in equation (S1.1d) are of size λ^{-1}, i.e. $\lambda \boldsymbol{\nabla}_y^2 \phi_1 \sim \mathcal{O}\left(\lambda^{-1}\right)$, and $\lambda^{-1} f''(\phi_0)\phi_1 \sim \mathcal{O}\left(\lambda^{-1}\right)$. The corresponding boundary conditions are,

$$\boldsymbol{u}_0 = 0, \qquad \boldsymbol{y} \in \partial B, \qquad \text{(S1.3}a\text{)}$$

$$\hat{\boldsymbol{n}} \cdot (\boldsymbol{\nabla}_x \phi_0 + \boldsymbol{\nabla}_y \phi_1) = |\boldsymbol{\nabla}_y \phi_1 + \boldsymbol{\nabla}_x \phi_0| \cos[\theta(\bar{C})], \qquad \boldsymbol{y} \in \partial B \qquad \text{(S1.3}b\text{)}$$

$$\hat{\boldsymbol{n}} \cdot \frac{M_0}{\eta_0} \boldsymbol{\nabla}_x \mu_0 + \hat{\boldsymbol{n}} \cdot \frac{M_0}{\eta_0} \boldsymbol{\nabla}_y \mu_1 = 0, \qquad \boldsymbol{y} \in \partial B. \qquad \text{(S1.3}c\text{)}$$

The exudate transport equation at $\mathcal{O}(\varepsilon^0)$ is

$$\frac{\partial c_0}{\partial \tau_0} + \frac{\partial c_1}{\partial \tau_{-1}} + \boldsymbol{u}_0 \cdot \boldsymbol{\nabla}_y c_0 - \boldsymbol{\nabla}_x \cdot D \left(\phi_0 \boldsymbol{\nabla}_y c_0 - c_0 \boldsymbol{\nabla}_y \phi_0 \right)$$
$$- \boldsymbol{\nabla}_y \cdot D \left(\phi_0 \boldsymbol{\nabla}_y c_1 + \phi_0 \boldsymbol{\nabla}_x c_0 + \phi_1 \boldsymbol{\nabla}_y c_0 - c_0 \boldsymbol{\nabla}_y \phi_1 - c_0 \boldsymbol{\nabla}_x \phi_0 - c_1 \boldsymbol{\nabla}_y \phi_0 \right) = 0, \; \boldsymbol{y} \in B, \qquad \text{(S1.3}d\text{)}$$

with boundary condition

$$\boldsymbol{n} \cdot D \left(\phi_0 \boldsymbol{\nabla}_y c_1 + \phi_0 \boldsymbol{\nabla}_x c_0 + \phi_1 \boldsymbol{\nabla}_y c_0 - c_0 \boldsymbol{\nabla}_y \phi_1 - c_0 \boldsymbol{\nabla}_x \phi_0 - c_1 \boldsymbol{\nabla}_y \phi_0 \right) = 0, \; \boldsymbol{y} \in \partial B. \qquad \text{(S1.3}e\text{)}$$

To check for the solvability of the equations, we start by integrating equation (S1.1a) over B and apply the divergence theorem,

$$\int_B \left[\frac{\partial \phi_0}{\partial \tau_0} + \frac{\partial \phi_1}{\partial \tau_{-1}} \right] d\boldsymbol{y} + \int_{\partial B} \left[\boldsymbol{u}_0 \cdot \hat{\boldsymbol{n}} \phi_0 - \Upsilon \left(\hat{\boldsymbol{n}} \cdot \frac{M_0}{\eta_0} \boldsymbol{\nabla}_y \mu_1 + \hat{\boldsymbol{n}} \cdot \frac{M_0}{\eta_0} \boldsymbol{\nabla}_x \mu_0 \right) \right] d\boldsymbol{y} = 0. \qquad \text{(S1.4)}$$

Using equations (S1.1c), (S1.3c) and (S1.3a), we find that for a solution to exist we need,

$$\int_B \left[\frac{\partial \phi_0}{\partial \tau_0} + \frac{\partial \phi_1}{\partial \tau_{-1}} \right] d\boldsymbol{y} = 0. \qquad \text{(S1.5)}$$

Integrating with respect to τ_{-1} between 0 and T_{-1} where $T_{-1} \gg 1$ such that ϕ_0 has been at steady state for sufficiently long time that $T_{-1} \gtrsim \mathcal{O}(\varepsilon^{-1})$, so $\frac{\partial \phi_0}{\partial \tau_{-1}} \approx 0$,

$$\int_B T_{-1} \frac{\partial \phi_0(T_{-1}, \tau_0, \ldots)}{\partial \tau_0} + \phi_1(T_{-1}, \tau_0, \ldots) - \phi_1(0, \tau_0, \ldots) \, d\boldsymbol{y} = 0 \qquad \text{(S1.6)}$$

So that ϕ_1 does not grow linearly with time we must have $\int_B \frac{\partial \phi_0}{\partial \tau_0}\, d\boldsymbol{y} = 0$. Therefore S, and hence ϕ_0, are independent of τ_0 and $\int_B \phi_1\, d\boldsymbol{y}$ is independent of τ_{-1}. As the equations for ϕ_1 are linear and as we are only interested in the limit $\tau_{-1} \to \infty$, we consider only the steady state case $\frac{\partial \phi_1}{\partial \tau_{-1}} = 0$.

Next, to check for the solvability of the transport equation, we integrate equation (S1.3*d*) over B and apply the divergence theorem,

$$\int_B \left[\frac{\partial c_0}{\partial \tau_0} + \frac{\partial c_1}{\partial \tau_{-1}} - \boldsymbol{\nabla}_x \cdot D\left(\phi_0 \boldsymbol{\nabla}_y c_0 - c_0 \boldsymbol{\nabla}_y \phi_0\right)\right] d\boldsymbol{y} + \int_{\partial B} \boldsymbol{u}_0 \cdot \hat{\boldsymbol{n}} c_0\, d\boldsymbol{y} \tag{S1.7}$$
$$+ \int_{\partial B} \hat{\boldsymbol{n}} \cdot \left(\phi_0 \boldsymbol{\nabla}_y c_1 + \phi_0 \boldsymbol{\nabla}_x c_0 + \phi_1 \boldsymbol{\nabla}_y c_0 - c_0 \boldsymbol{\nabla}_y \phi_1 - c_0 \boldsymbol{\nabla}_x \phi_0 - c_1 \boldsymbol{\nabla}_y \phi_0\right)\, d\boldsymbol{y} = 0$$

Using equation (S1.3*e*) and (S1.3*a*), substituting $c_0 = \bar{C}\phi_0$, and using the knowledge that ϕ_0 is independent of τ_0, $\bar{C}$ and ϕ_1 are independent of τ_{-1}, we find that for a solution to exist it is required that,

$$\int_B \left[\phi_0 \frac{\partial \bar{C}}{\partial \tau_0} + \frac{\partial c_1}{\partial \tau_{-1}}\right] d\boldsymbol{y} = 0. \tag{S1.8}$$

We integrate with respect to τ_{-1} between 0 and T_{-1},

$$T_{-1} \int_B \phi_0 \frac{\partial \bar{C}}{\partial \tau_0}\, d\boldsymbol{y} + \int_B \phi_0 \left[c_1(T_{-1}, \tau_0, ...) - c_1(0, \tau_0, ...)\right]\, d\boldsymbol{y} = 0 \tag{S1.9}$$

For c_1 not to grow linearly in time we need $\int_B \phi_0 \frac{\partial \bar{C}}{\partial \tau_0}\, d\boldsymbol{y} = 0$. Since ϕ_0 is not 0 everywhere and $\bar{C} \sim \bar{C}(\boldsymbol{x})$, $\bar{C}$ must be independent of τ_0 and therefore $\int_B c_1\, d\boldsymbol{y}$ is independent of τ_{-1}. Again, we are interested in the limit $\tau_{-1} \to 0$. As equations (S1.3*d*) are linear in c_1 we solve for steady state only. Therefore, substituting $c_0 = \bar{C}\phi_0$ into equations (S1.3*d*) and (S1.3*e*) we get,

$$\begin{aligned} -\bar{C}\boldsymbol{u}_0 \cdot \boldsymbol{\nabla}_y \phi_0 + \boldsymbol{\nabla}_y \cdot \left[\phi_0 \boldsymbol{\nabla}_y c_1 - c_1 \boldsymbol{\nabla}_y \phi_0 + \bar{C}\left(\phi_1 \boldsymbol{\nabla}_y \phi_0 - \phi_0 \boldsymbol{\nabla}_y \phi_1\right)\right] \\ = -\boldsymbol{\nabla}_y \cdot \left(\phi_0^2 \boldsymbol{\nabla}_x \bar{C} + \phi_0 \bar{C} \boldsymbol{\nabla}_x \phi_0\right), \end{aligned} \qquad \boldsymbol{y} \in B, \tag{S1.10a}$$

$$\hat{\boldsymbol{n}} \cdot \left[\phi_0 \boldsymbol{\nabla}_y c_1 - c_1 \boldsymbol{\nabla}_y \phi_0 + \bar{C}\left(\phi_1 \boldsymbol{\nabla}_y \phi_0 - \phi_0 \boldsymbol{\nabla}_y \phi_1\right)\right] = -\hat{\boldsymbol{n}} \cdot \left(\phi_0^2 \boldsymbol{\nabla}_x \bar{C} + \phi_0 \bar{C} \boldsymbol{\nabla}_x \phi_0\right), \quad \boldsymbol{y} \in \partial B. \tag{S1.10b}$$

From these equations we can see that c_1 is dependent on ϕ_1. Equations (S1.1) and (S1.3), for ϕ_0 at steady state, reduce to,

$$\boldsymbol{u}_0 \cdot \boldsymbol{\nabla}_y \phi_0 = \Upsilon \left(\boldsymbol{\nabla}_y \cdot \frac{M_0}{\eta_0} \boldsymbol{\nabla}_y \mu_1 + \boldsymbol{\nabla}_y \cdot \frac{M_0}{\eta_0} \boldsymbol{\nabla}_x \mu_0\right), \qquad \boldsymbol{y} \in B, \tag{S1.11a}$$

$$\boldsymbol{\nabla}_y \cdot \eta_0 \boldsymbol{\sigma}_{0y} - \boldsymbol{\nabla}_x p_0 - \boldsymbol{\nabla}_y p_1 - \phi_0 \boldsymbol{\nabla}_y \mu_1 - \phi_0 \boldsymbol{\nabla}_x \mu_0 = \phi_0 g \hat{\boldsymbol{e}}_3, \qquad \boldsymbol{y} \in B, \tag{S1.11b}$$

$$\boldsymbol{\nabla}_y \cdot \boldsymbol{u}_0 = 0, \qquad \boldsymbol{y} \in B, \tag{S1.11c}$$

$$\mu_1 = \gamma(\bar{C}) \left[\lambda^{-1} f''(\phi_0)\phi_1 - \lambda \boldsymbol{\nabla}_y^2 \phi_1\right], \qquad \boldsymbol{y} \in B, \tag{S1.11d}$$

where the boundary conditions are,

$$\boldsymbol{u}_0 = 0, \qquad \boldsymbol{y} \in \partial B, \tag{S1.11e}$$

$$\hat{\boldsymbol{n}} \cdot \boldsymbol{\nabla}_x \phi_0 + \hat{\boldsymbol{n}} \cdot \boldsymbol{\nabla}_y \phi_1 = |\boldsymbol{\nabla}_y \phi_1 + \boldsymbol{\nabla}_x \phi_0| \cos[\theta(\bar{C})], \qquad \boldsymbol{y} \in \partial B, \tag{S1.11f}$$

$$\hat{\boldsymbol{n}} \cdot \frac{M_0}{\eta_0} \boldsymbol{\nabla}_x \mu_0 + \hat{\boldsymbol{n}} \cdot \frac{M_0}{\eta_0} \boldsymbol{\nabla}_y \mu_1 = 0, \qquad \boldsymbol{y} \in \partial B., \tag{S1.11g}$$

Before we proceed it is useful to estimate the size of ϕ_1 in equations (S1.10), which we will show can be neglected from the analysis. We start by considering the size of ϕ_0, which defines the location of the air–water interface. We know that ϕ_0 is constant far from the interface and varies

rapidly over a distance λ at the interface. Hence, we consider the size of ϕ_1 both close to and far from the interface.

Far from the interface ϕ_0 takes values $\phi_0 = 0 + \mathcal{O}(\lambda)$, or $\phi_0 = 1 + \mathcal{O}(\lambda)$. Hence, $M_0 = \phi_0^2(1-\phi_0)^2 \sim O(\lambda^2)$ and $\nabla_y \phi_0 = 0$. Hence, equations (S1.11*a*) and (S1.11*g*) simplify to

$$\nabla_y \cdot \left[\frac{M_0}{\eta_0}(\nabla_y \mu_1 + \nabla_x \mu_0)\right] = 0, \qquad \boldsymbol{y} \in B, \tag{S1.12a}$$

with boundary condition

$$\hat{\boldsymbol{n}} \cdot \left[\frac{M_0}{\eta_0}(\nabla_y \mu_1 + \nabla_x \mu_0)\right] = 0, \qquad \boldsymbol{y} \in \partial B. \tag{S1.12b}$$

From which we conclude that μ_1 is driven by the terms $\nabla_x \mu_0$ and is of order 1. Using equation (S1.11*d*) and $f''(\phi_0) = 12\phi_0^2 - 12\phi_0 + 2 = 2 + O(\lambda)$, we find $\phi_1 \sim \mathcal{O}(\lambda)$. Therefore, neglecting terms of size $\lesssim \mathcal{O}(\lambda)$, equations (S1.10) can be written

$$\nabla_y \cdot (\phi_0 \nabla_y c_1 - c_1 \nabla_y \phi_0) = -\nabla_y \cdot \left(\phi_0^2 \nabla_x \bar{C}\right), \qquad \boldsymbol{y} \in B, \tag{S1.13a}$$

$$\hat{\boldsymbol{n}} \cdot (\phi_0 \nabla_y c_1 - c_1 \nabla_y \phi_0) = -\hat{\boldsymbol{n}} \cdot \phi_0^2 \nabla_x \bar{C}, \qquad \boldsymbol{y} \in \partial B. \tag{S1.13b}$$

Here we have noticed that, in the absence of Haines jumps, $\frac{\delta\phi_0}{\delta S} = 0$ far from the interface, *i.e.*, a small change in saturation causes the interface to move a small amount. Hence, we conclude that, in the limit $\lambda \to 0$, the ϕ_1 terms play no role in the calculation of c_1 far from the interface.

Close to the interface ϕ_0 varies on a scale λ, hence, we rescale $\nabla_y = \frac{1}{\lambda}\check{\nabla}_y$ and, substituting into equation (S1.11*a*) we find

$$\lambda^{-1}\boldsymbol{u}_0 \cdot \check{\nabla}_y \phi_0 = \Upsilon\left(\lambda^{-2}\check{\nabla}_y \cdot \frac{M_0}{\eta_0}\check{\nabla}_y \mu_1 + \lambda^{-1}\check{\nabla}_y \cdot \frac{M_0}{\eta_0}\nabla_x \mu_0\right), \qquad \boldsymbol{y} \in B. \tag{S1.14}$$

We find a balance if $\mu_1 \sim \mathcal{O}(\lambda)$, so we write $\mu_1 = \lambda\bar{\mu}_1$. Next, we consider equation (S1.11*d*), rescaled close to the interface and we find,

$$\lambda^2 \bar{\mu}_1 = f''(\phi_0)\phi_1 - \check{\nabla}_y^2 \phi_1, \qquad \boldsymbol{x} \in B, \tag{S1.15a}$$

with the boundary condition

$$\hat{\boldsymbol{n}} \cdot (\nabla_x \phi_0 + \lambda^{-1}\check{\nabla}_y \phi_1) = |\nabla_x \phi_0 + \lambda^{-1}\check{\nabla}_y \phi_1| \cos\theta, \qquad \boldsymbol{y} \in \partial B. \tag{S1.15b}$$

Using the observation $\phi_0 = \phi_0[\boldsymbol{y}, S(\boldsymbol{x})]$ we can write

$$\nabla_x \phi_0 = \frac{\delta\phi_0}{\delta S}\nabla_x S, \tag{S1.16}$$

which is $\mathcal{O}(1)$ close to the interface. Hence, the terms containing $\nabla_x \phi_0$ in equation (S1.15*b*) are the largest source terms in equations (S1.15). Equations (S1.15) are linear so $\phi_1 \sim \mathcal{O}(\lambda)$, substituting into equations (S1.10)

$$-\lambda^{-1}\bar{C}\boldsymbol{u}_0 \cdot \check{\nabla}_y \phi_0 + \lambda^{-2}\check{\nabla}_y \cdot (\phi_0 \check{\nabla}_y c_1 - c_1 \check{\nabla}_y \phi_0) = 0, \qquad \boldsymbol{y} \in B \tag{S1.17}$$

$$\hat{\boldsymbol{n}} \cdot \lambda^{-1}(\phi_0 \check{\nabla}_y c_1 - c_1 \check{\nabla}_y \phi_0) = 0, \qquad \boldsymbol{y} \in \partial B. \tag{S1.18}$$

In order to balance this equation (S1.17), either the velocity must be scaled with λ^{-1}, i.e. $\boldsymbol{u}_0 = \lambda^{-1}\bar{\boldsymbol{u}}_0$, which would imply that the velocity becomes large as $\lambda \to 0$, or the velocity term is of $\mathcal{O}(1)$ and so as $\lambda \to 0$, we find,

$$\nabla_y \cdot (\phi_0 \check{\nabla}_y c_1 - c_1 \check{\nabla}_y \phi_0) = 0, \qquad \boldsymbol{y} \in B \tag{S1.19}$$

$$\hat{\boldsymbol{n}} \cdot (\phi_0 \check{\nabla}_y c_1 - c_1 \check{\nabla}_y \phi_0) = 0, \qquad \boldsymbol{y} \in \partial B, \tag{S1.20}$$

and the ϕ_1 terms in equations (S1.10) play no role in the calculation of c_1 close to the interface.

Since neither the ϕ_1 terms nor the velocity term drive c_1 either close to or far from the interface they can be neglected from equations (S1.10). Hence, the transport equations decouple from the order 1 phase terms and reduce to an equation for c_1,

$$\nabla_y \cdot (\phi_0 \nabla_y c_1 - c_1 \nabla_y \phi_0) = -\nabla_y \cdot \left(\phi_0^2 \nabla_x \bar{C}\right), \qquad \boldsymbol{y} \in B, \tag{S1.21a}$$

with boundary condition

$$\hat{\boldsymbol{n}} \cdot (\phi_0 \nabla_y c_1 - c_1 \nabla_y \phi_0) = -\hat{\boldsymbol{n}} \cdot \phi_0^2 \nabla_x \bar{C}, \qquad \boldsymbol{y} \in \partial B. \tag{S1.21b}$$

To solve equations (S1.11), we look for solutions in separable form,

$$\boldsymbol{u}_0 = \sum_k \boldsymbol{\kappa}_k^\mu(\boldsymbol{y}) \frac{\partial_{x_k} \mu_0(\boldsymbol{x})}{\eta(\bar{C}(\boldsymbol{x}))} + \boldsymbol{\kappa}_k^p(\boldsymbol{y}) \frac{\partial_{x_k} p_0(\boldsymbol{x})}{\eta(\bar{C}(\boldsymbol{x}))} + \boldsymbol{\kappa}^g(\boldsymbol{y}) \frac{g}{\eta(\bar{C}(\boldsymbol{x}))}, \tag{S1.22a}$$

$$\mu_1 = \sum_k \chi_k^\mu(\boldsymbol{y}) \partial_{x_k} \mu_0(\boldsymbol{x}) + \chi_k^p(\boldsymbol{y}) \partial_{x_k} p_0(\boldsymbol{x}) + \chi^g(\boldsymbol{y}) g, \tag{S1.22b}$$

$$p_1 = \sum_k \omega_k^\mu(\boldsymbol{y}) \partial_{x_k} \mu_0(\boldsymbol{x}) + \omega_k^p(\boldsymbol{y}) \partial_{x_k} p_0(\boldsymbol{x}) + \omega^g(\boldsymbol{y}) g, \tag{S1.22c}$$

$$\phi_1 = \frac{1}{\gamma(\bar{C}(\boldsymbol{x}))} \sum_k \psi_k^\mu(\boldsymbol{y}) \partial_{x_k} \mu_0(\boldsymbol{x}) + \psi_k^p(\boldsymbol{y}) \partial_{x_k} p_0(\boldsymbol{x}) + \psi^g(\boldsymbol{y}) g, \tag{S1.22d}$$

$$c_1 = \sum_k \xi^c(\boldsymbol{y}) \partial_{x_k} \bar{C}(\boldsymbol{x}). \tag{S1.22e}$$

We substitute the solutions in separable form, equations (S1.22), into equations (S1.11) and gather terms dependent on μ_0 to get,

$$\boldsymbol{\kappa}_k^\mu \cdot \nabla_y \phi_0 = \Upsilon \left(\nabla_y \cdot \frac{M_0}{\eta_0^\phi} \nabla_y \chi_k^\mu + \nabla_y \cdot \frac{M_0}{\eta_0^\phi} \hat{\boldsymbol{e}}_k \right), \qquad \boldsymbol{y} \in B, \tag{S1.23a}$$

$$\nabla_y \cdot \eta_0^\phi \boldsymbol{\sigma}_{0y}^\mu - \nabla_y \omega_k^\mu - \phi_0 \nabla_y \chi_k^\mu - \phi_0 \hat{\boldsymbol{e}}_k = 0, \qquad \boldsymbol{y} \in B, \tag{S1.23b}$$

$$\nabla_y \cdot \boldsymbol{\kappa}_k^\mu = 0, \qquad \boldsymbol{y} \in B, \tag{S1.23c}$$

$$\chi_k^\mu = \lambda^{-1} f''(\phi_0) \psi_k^\mu - \lambda \nabla_y^2 \psi_k^\mu, \qquad \boldsymbol{y} \in B, \tag{S1.23d}$$

where $\boldsymbol{\sigma}_{0y}^\mu = \nabla_y \boldsymbol{\kappa}_k^\mu + \left(\nabla_y \boldsymbol{\kappa}_k^\mu\right)^T$. The corresponding boundary conditions are,

$$\boldsymbol{\kappa}_k^\mu = 0, \qquad \boldsymbol{y} \in \partial B, \tag{S1.23e}$$

$$\hat{\boldsymbol{n}} \cdot \nabla_y \psi_k^\mu = \left|\nabla_y \psi_k^\mu\right| \cos[\theta(\bar{C})], \qquad \boldsymbol{y} \in \partial B, \tag{S1.23f}$$

$$\hat{\boldsymbol{n}} \cdot M_0 \hat{\boldsymbol{e}}_k + \hat{\boldsymbol{n}} \cdot M_0 \nabla_y \chi_k^\mu = 0, \qquad \boldsymbol{y} \in \partial B. \tag{S1.23g}$$

Equations (S1.23) determine the fluid velocity driven by a large scale variation in capillary pressure. Physically, this corresponds to the difference in pressure between the two phases. Hence, in the limit $\lambda \to 0$, only the water phase is directly driven. The air phase is not directly driven, but can be set in motion by the water velocity at the air–water boundary.

Next, we gather terms dependent on p_0,

$$\boldsymbol{\kappa}_k^p \cdot \nabla_y \phi_0 = \Upsilon \left(\nabla_y \cdot \frac{M_0}{\eta_0^\phi} \nabla_y \chi_k^p \right), \qquad \boldsymbol{y} \in B, \tag{S1.24a}$$

$$\nabla_y \cdot \eta_0^\phi \boldsymbol{\sigma}_{0y}^p - \hat{\boldsymbol{e}}_k - \nabla_y \omega_k^p - \phi_0 \nabla_y \chi_k^p = 0, \qquad \boldsymbol{y} \in B, \tag{S1.24b}$$

$$\nabla_y \cdot \boldsymbol{\kappa}_k^p = 0, \qquad \boldsymbol{y} \in B, \tag{S1.24c}$$

$$\chi_k^p = \lambda^{-1} f''(\phi_0) \psi_k^p - \lambda \nabla_y^2 \psi_k^p, \qquad \boldsymbol{y} \in B, \tag{S1.24d}$$

where $\boldsymbol{\sigma}^p_{0y} = \boldsymbol{\nabla}_y \boldsymbol{\kappa}^p_k + \left(\boldsymbol{\nabla}_y \boldsymbol{\kappa}^p_k\right)^T$. The corresponding boundary conditions are,

$$\boldsymbol{\kappa}^p_k = 0, \qquad \boldsymbol{y} \in \partial B, \tag{S1.24e}$$

$$\hat{\boldsymbol{n}} \cdot \boldsymbol{\nabla}_y \psi^p_k = \left|\boldsymbol{\nabla}_y \psi^p_k\right| \cos[\theta(\bar{C})], \qquad \boldsymbol{y} \in \partial B, \tag{S1.24f}$$

$$\hat{\boldsymbol{n}} \cdot M_0 \boldsymbol{\nabla}_y \chi^p_k = 0, \qquad \boldsymbol{y} \in \partial B. \tag{S1.24g}$$

Equations (S1.24) determine the fluid velocity due to a unit pressure gradient, in this case both the air and water phases are driven by the combined pressure p_0.

Next, we gather terms dependent on g,

$$\boldsymbol{\kappa}^g \cdot \boldsymbol{\nabla}_y \phi_0 = \Upsilon \left(\boldsymbol{\nabla}_y \cdot \frac{M_0}{\eta^\phi_0} \boldsymbol{\nabla}_y \chi^g \right), \qquad \boldsymbol{y} \in B, \tag{S1.25a}$$

$$\boldsymbol{\nabla}_y \cdot \eta^\phi_0 \boldsymbol{\sigma}^g_{0y} - \boldsymbol{\nabla}_y \omega^g - \phi_0 \boldsymbol{\nabla}_y \chi^g = \phi_0 \hat{\boldsymbol{e}}_k, \qquad \boldsymbol{y} \in B, \tag{S1.25b}$$

$$\boldsymbol{\nabla}_y \cdot \boldsymbol{\kappa}^g = 0, \qquad \boldsymbol{y} \in B, \tag{S1.25c}$$

$$\chi^g = \lambda^{-1} f''(\phi_0) \psi^g - \lambda \boldsymbol{\nabla}^2_y \psi^g, \qquad \boldsymbol{y} \in B, \tag{S1.25d}$$

where $\boldsymbol{\sigma}^g_{0y} = \boldsymbol{\nabla}_y \boldsymbol{\kappa}^g + (\boldsymbol{\nabla}_y \boldsymbol{\kappa}^g)^T$. The corresponding boundary conditions are,

$$\boldsymbol{\kappa}^g = 0, \qquad \boldsymbol{y} \in \partial B, \tag{S1.25e}$$

$$\hat{\boldsymbol{n}} \cdot \boldsymbol{\nabla}_y \psi^g = \left|\boldsymbol{\nabla}_y \psi^g\right| \cos[\theta(\bar{C})], \qquad \boldsymbol{y} \in \partial B, \tag{S1.25f}$$

$$\hat{\boldsymbol{n}} \cdot M_0 \boldsymbol{\nabla}_y \chi^g = 0, \qquad \boldsymbol{y} \in \partial B. \tag{S1.25g}$$

Equations (S1.25) determine the fluid velocity due to gravity. As the water density is much larger than the air density, only the water phase is directly driven. Any induced movement of the air phase comes from the effect of water movement at the air–water interface.

Finally, we gather terms dependent on c_0,

$$\boldsymbol{\nabla}_y \cdot \left(\phi_0 \boldsymbol{\nabla}_y \xi^c_k - \xi^c_k \boldsymbol{\nabla}_y \phi_0\right) = -\boldsymbol{\nabla}_y \cdot \left(\phi_0^2 \hat{\boldsymbol{e}}_k\right), \qquad \boldsymbol{y} \in B, \tag{S1.26a}$$

with boundary condition

$$\hat{\boldsymbol{n}} \cdot \left(\phi_0 \boldsymbol{\nabla}_y \xi^c_k - \xi^c_k \nabla_y \phi_0\right) = -\hat{\boldsymbol{n}} \cdot \phi_0^2 \hat{\boldsymbol{e}}_k, \qquad \boldsymbol{y} \in \partial B. \tag{S1.26b}$$

Equations (S1.26) determine the local impedance to diffusion offered by the soil structure and the position of the air water interface. Physically, this will be combined with the unimpeded diffusion coefficient to calculate the effective diffusion coefficient in the water phase as a function of saturation.

Cell problems (S1.23), (S1.24), (S1.25) and (S1.26), for known $\theta(\bar{C})$, provide a complete description of how the pore scale geometry and physical processes are dependent on large scale variations in capillary pressure, combined pressure, acceleration due to gravity and the concentration of root exudates. The cell problems (S1.23) to (S1.26) are equivalent to the cell problems (2.19) to (2.22) presented in the main text.

(b) $\mathcal{O}(\varepsilon^1)$

We expand the phase equation (2.8*a*) and conservation equation (2.8*c*) at order ε^1 using expansions (S1.22) with $\nabla_y \mu_0 = 0$,

$$\frac{\partial \phi_0}{\partial \tau_1} + \frac{\partial \phi_1}{\partial \tau_0} + \frac{\partial \phi_2}{\partial \tau_{-1}} + \boldsymbol{u}_1 \cdot \nabla_y \phi_0 + \boldsymbol{u}_0 \cdot \nabla_y \phi_1 + \boldsymbol{u}_0 \cdot \nabla_x \phi_0$$
$$- \Upsilon \left(\nabla_y \cdot \frac{M_0}{\eta_0} (\nabla_y \mu_2 + \nabla_x \mu_1) + \nabla_y \cdot \frac{M_1}{\eta_0} (\nabla_y \mu_1 + \nabla_x \mu_0) \right) \tag{S1.27a}$$
$$- \Upsilon \left(-\nabla_y \cdot \frac{\eta_1 M_0}{\eta_0^2} (\nabla_y \mu_1 + \nabla_x \mu_0) + \nabla_x \cdot \frac{M_0}{\eta_0} (\nabla_y \mu_1 + \nabla_x \mu_0) \right) = 0, \quad \boldsymbol{y} \in B$$

and

$$\nabla_x \cdot \boldsymbol{u}_0 + \nabla_y \cdot \boldsymbol{u}_1 = 0, \qquad \boldsymbol{y} \in B, \tag{S1.27b}$$

where ϕ_2 and μ_2 are periodic with period 1. The corresponding boundary conditions, equations (2.8*e*) and (2.8*g*), are also expanded at order ε^1 using expansions (S1.22),

$$\boldsymbol{u}_1 = 0, \qquad \boldsymbol{y} \in \partial B \tag{S1.28a}$$

$$\hat{\boldsymbol{n}} \cdot \frac{M_0}{\eta_0} (\nabla_y \mu_2 + \nabla_x \mu_1) + \hat{\boldsymbol{n}} \cdot \frac{M_1}{\eta_0} (\nabla_y \mu_1 + \nabla_x \mu_0)$$
$$- \hat{\boldsymbol{n}} \cdot \frac{\eta_1 M_0}{\eta_0^2} (\nabla_y \mu_1 + \nabla_x \mu_0) = 0, \qquad \boldsymbol{y} \in \partial B. \tag{S1.28b}$$

To check the solvability of equation (S1.27*a*), we integrate over B and apply the divergence theorem,

$$\int_B \left[\frac{\partial \phi_0}{\partial \tau_1} + \frac{\partial \phi_1}{\partial \tau_0} + \frac{\partial \phi_2}{\partial \tau_{-1}} + \nabla_x \cdot (\boldsymbol{u}_0 \phi_0) - \phi_0 \nabla_y \cdot \boldsymbol{u}_1 - \phi_1 \nabla_y \cdot \boldsymbol{u}_0 - \phi_0 \nabla_x \cdot \boldsymbol{u}_0 \right] d\boldsymbol{y}$$
$$- \Upsilon \int_{\partial B} \hat{\boldsymbol{n}} \cdot \left[\frac{M_0}{\eta_0} (\nabla_y \mu_2 + \nabla_x \mu_1) + \frac{M_1}{\eta_0} (\nabla_y \mu_1 + \nabla_x \mu_0) - \frac{\eta_1 M_0}{\eta_0^2} (\nabla_y \mu_1 + \nabla_x \mu_0) \right] d\boldsymbol{y} \tag{S1.29}$$
$$+ \int_{\partial B} \hat{\boldsymbol{n}} \cdot [(\boldsymbol{u}_1 \phi_0) + (\boldsymbol{u}_0 \phi_1)] \, d\boldsymbol{y} - \Upsilon \int_B \left[\nabla_x \cdot \frac{M_0}{\eta_0} (\nabla_y \mu_1 + \nabla_x \mu_0) \right] d\boldsymbol{y} = 0, \qquad \boldsymbol{y} \in B.$$

Equations (S1.28*b*), (S1.28*a*), (S1.3*a*), (S1.11*c*) and (S1.27*b*) allow us to set many of the terms to zero, so equation (S1.30) reduces to,

$$\int_B \left[\frac{\partial \phi_0}{\partial \tau_1} + \frac{\partial \phi_1}{\partial \tau_0} + \frac{\partial \phi_2}{\partial \tau_{-1}} + \nabla_x \cdot (\boldsymbol{u}_0 \phi_0) - \Upsilon \nabla_x \cdot \frac{M_0}{\eta_0} (\nabla_y \mu_1 + \nabla_x \mu_0) \right] d\boldsymbol{y} = 0. \tag{S1.30}$$

Next we integrate with respect to τ_{-1} between 0 and $T_{-1} \gg 1$, such that ϕ_0 has been at steady state for sufficiently long time,

$$\int_B [\phi_2(T_{-1}, \tau_0, \tau_1) - \phi_2(0, \tau_0, \tau_1)] \, d\boldsymbol{y}$$
$$+ T_{-1} \int_B \left[\frac{\partial \phi_0(T_{-1}, \tau_0, \tau_1)}{\partial \tau_1} + \frac{\partial \phi_1(\tau_0, \tau_1)}{\partial \tau_0} + \nabla_x \cdot (\boldsymbol{u}_0 \phi_0) \right] d\boldsymbol{y} \tag{S1.31}$$
$$- T_{-1} \int_B \left[\Upsilon \nabla_x \cdot \frac{M_0}{\eta_0} (\nabla_y \mu_1 + \nabla_x \mu_0) \right] d\boldsymbol{y} = 0.$$

So that ϕ_2 does not grow linearly with time it must be true that,

$$\int_B \left[\frac{\partial \phi_0(T_{-1}, \tau_0, \tau_1)}{\partial \tau_1} + \frac{\partial \phi_1(\tau_0, \tau_1)}{\partial \tau_0} + \nabla_x \cdot (\boldsymbol{u}_0 \phi_0) \right] d\boldsymbol{y} \tag{S1.32}$$
$$- T_{-1} \int_B \left[\Upsilon \nabla_x \cdot \frac{M_0}{\eta_0} (\nabla_y \mu_1 + \nabla_x \mu_0) \right] d\boldsymbol{y} = 0,$$

and therefore ϕ_2 must be independent of τ_{-1}. This gives us the average effect of processes occurring over the fastest time scale. We integrate again, this time with respect to τ_0 between 0 and $T_0 \gg 1$ so that ϕ_0 has been at steady state for sufficiently long time,

$$\int_B [\phi_1(T_0,\tau_1) - \phi_1(0,\tau_1)]\ d\boldsymbol{y} \qquad \text{(S1.33)}$$
$$+ T_0 \int_B \left[\frac{\partial \phi_0(\tau_{-1},T_0,\tau_1)}{\partial \tau_1} + \boldsymbol{\nabla}_x \cdot (\boldsymbol{u}_0\phi_0) - \Upsilon \boldsymbol{\nabla}_x \cdot \frac{M_0}{\eta_0}\left(\boldsymbol{\nabla}_y \mu_1 + \boldsymbol{\nabla}_x \mu_0\right)\right]\ d\boldsymbol{y} = 0.$$

So that ϕ_1 does not grow linearly with time is must hold that,

$$\int_B \left[\frac{\partial \phi_0(\tau_{-1},T_0,\tau_1)}{\partial \tau_1} + \boldsymbol{\nabla}_x \cdot (\boldsymbol{u}_0\phi_0) - \Upsilon \boldsymbol{\nabla}_x \cdot \frac{M_0}{\eta_0}\left(\boldsymbol{\nabla}_y \mu_1 + \boldsymbol{\nabla}_x \mu_0\right)\right]\ d\boldsymbol{y} = 0, \qquad \text{(S1.34)}$$

and therefore ϕ_1 must be independent of τ_0. This gives us the average effect of process occurring over the second fastest time scale.

For the next step, we take the limit as $\lambda \to 0$, i.e. the limit at which the water-air interface becomes infinitely thin, which implies $\int_B M_0\ d\boldsymbol{y} \sim \lambda \to 0$, since M_0 is only non-zero within a region of width λ. In this limit equation (S1.34), reduces to,

$$\int_B \left[\frac{\partial \phi_0(\tau_{-1},T_0,\tau_1)}{\partial \tau_1} + \boldsymbol{\nabla}_x \cdot (\boldsymbol{u}_0\phi_0)\right]\ d\boldsymbol{y} = 0, \qquad \text{(S1.35)}$$

We then substitute in the series solution for $\boldsymbol{u}_0$,

$$\|B\|\frac{\partial S}{\partial \tau_1} + \boldsymbol{\nabla}_x \cdot \left(\int_B \left[\phi_0 \boldsymbol{\kappa}_k^{\mu} \otimes \hat{\boldsymbol{e}}_k\right]\ d\boldsymbol{y} \frac{\boldsymbol{\nabla}_x \mu_0}{\eta(\bar{C})} + \int_B \left[\phi_0 \boldsymbol{\kappa}_k^{p} \otimes \hat{\boldsymbol{e}}_k\right]\ d\boldsymbol{y} \frac{\boldsymbol{\nabla}_x p_0}{\eta(\bar{C})}\right) \qquad \text{(S1.36)}$$
$$+ \boldsymbol{\nabla}_x \cdot \left(\int_B \left[\phi_0 \boldsymbol{\kappa}^{g} \otimes \hat{\boldsymbol{e}}_3\right]\ d\boldsymbol{y} \frac{\hat{\boldsymbol{e}}_3 g}{\eta(\bar{C})}\right) = 0$$

This can be rewritten as

$$\|B\|\frac{\partial S}{\partial \tau_1} + \boldsymbol{\nabla}_x \cdot \boldsymbol{U} = 0, \qquad \text{(S1.37)}$$

where

$$\boldsymbol{U} = \frac{K[S,\theta(\bar{C})]}{\eta(\bar{C})}\boldsymbol{\nabla}_x \mu_0 + \frac{b[S,\theta(\bar{C})]}{\eta(\bar{C})}\boldsymbol{\nabla}_x p_0 + \frac{b_g[S,\theta(\bar{C})]}{\eta(\bar{C})}\hat{\boldsymbol{e}}_3 g. \qquad \text{(S1.38)}$$

This is equation (2.23*a*) from the main text. Equation (S1.37) tells us how the macroscale saturation varies with time depending on the concentration dependent viscosity and surface tension and is related to the pore scale behaviour by the parameters $K[S,\theta(\bar{C})]$, $b[S,\theta(\bar{C})]$ and $b_g[S,\theta(\bar{C})]$ that are defined as

$$K[S,\theta(\bar{C})] = \int_B \phi_0 \boldsymbol{\kappa}_k^{\mu} \otimes \hat{\boldsymbol{e}}_k\ d\boldsymbol{y}, \qquad \text{(S1.39}a\text{)}$$

$$b[S,\theta(\bar{C})] = \int_B \phi_0 \boldsymbol{\kappa}_k^{p} \otimes \hat{\boldsymbol{e}}_k\ d\boldsymbol{y}, \qquad \text{(S1.39}b\text{)}$$

$$b_g[S,\theta(\bar{C})] = \int_B \phi_0 \boldsymbol{\kappa}^{g} \otimes \hat{\boldsymbol{e}}_3\ d\boldsymbol{y}. \qquad \text{(S1.39}c\text{)}$$

Integrating equation (S1.27*b*) over the domain B yields,

$$\boldsymbol{\nabla}_x \cdot \bar{\boldsymbol{U}} = 0, \qquad \text{(S1.40)}$$

where

$$\bar{\boldsymbol{U}} = \frac{\bar{K}[S,\theta(\bar{C})]}{\eta(\bar{C})}\boldsymbol{\nabla}_x \mu_0 + \frac{\bar{b}[S,\theta(\bar{C})]}{\eta(\bar{C})}\boldsymbol{\nabla}_x p_0 + \frac{\bar{b}_g[S,\theta(\bar{C})]}{\eta(\bar{C})}\hat{\boldsymbol{e}}_3 g = 0. \qquad \text{(S1.41)}$$

rspa.royalsocietypublishing.org Proc R Soc A 0000000

This is equation (2.23*b*) from the main text and ensures that conservation of mass for the saturation equations where, again, the concentration dependent viscosity and surface tension are present and is related to the pore scale behaviour by,

$$\bar{K}[S,\theta(\bar{C})] = \int_B \boldsymbol{\kappa}_k^{\mu} \otimes \hat{\boldsymbol{e}}_k \, d\boldsymbol{y}, \tag{S1.42a}$$

$$\bar{b}[S,\theta(\bar{C})] = \int_B \boldsymbol{\kappa}_k^{p} \otimes \hat{\boldsymbol{e}}_k \, d\boldsymbol{y}, \tag{S1.42b}$$

$$\bar{b}_g[S,\theta(\bar{C})] = \int_B \boldsymbol{\kappa}^{g} \otimes \hat{\boldsymbol{e}}_3 \, d\boldsymbol{y}. \tag{S1.42c}$$

We now expand the transport equation (2.8*h*) with expansions (S1.22) to obtain

$$\begin{aligned}
&\phi_0\left(\frac{\partial c_0}{\partial \tau_1}+\frac{\partial c_1}{\partial \tau_0}+\frac{\partial c_2}{\partial \tau_{-1}}\right)+\phi_1\left(\frac{\partial c_0}{\partial \tau_0}+\frac{\partial c_1}{\partial \tau_{-1}}\right)+\phi_2\frac{\partial c_0}{\partial \tau_{-1}}\\
&+\boldsymbol{u}_1\cdot\nabla_y c_0+\boldsymbol{u}_0\cdot\nabla_y c_1+\boldsymbol{u}_0\cdot\nabla_x c_0-c_0\left(\frac{\partial \phi_0}{\partial \tau_1}+\frac{\partial \phi_1}{\partial \tau_0}+\frac{\partial \phi_2}{\partial \tau_{-1}}\right)\\
&-c_1\left(\frac{\partial \phi_0}{\partial \tau_0}+\frac{\partial \phi_1}{\partial \tau_{-1}}\right)-c_2\frac{\partial \phi_0}{\partial \tau_{-1}}-\nabla_y\cdot D\left(\phi_0\nabla_y c_2+\phi_0\nabla_x c_1+\phi_1\nabla_y c_1+\phi_1\nabla_x c_0\right)\\
&-\nabla_y\cdot D\left(\phi_2\nabla_y c_0-c_0\nabla_y\phi_2-c_0\nabla_x\phi_1-c_1\nabla_y\phi_1-c_1\nabla_x\phi_0-c_2\nabla_y\phi_0\right)\\
&-\nabla_x\cdot D\left(\phi_0\nabla_y c_1+\phi_0\nabla_x c_0+\phi_1\nabla_y c_0-c_0\nabla_y\phi_1-c_0\nabla_x\phi_0-c_1\nabla_y\phi_0\right)=0,
\end{aligned} \tag{S1.43}$$

and we expand the corresponding boundary condition, equation(2.8*i*) with expansions (S1.22),

$$\begin{aligned}
&\boldsymbol{n}\cdot D\left(\phi_0\nabla_y c_2+\phi_0\nabla_x c_1+\phi_1\nabla_y c_1+\phi_1\nabla_x c_0\right)\\
&+\boldsymbol{n}\cdot D\left(\phi_2\nabla_y c_0-c_0\nabla_y\phi_2-c_0\nabla_x\phi_1-c_1\nabla_y\phi_1-c_1\nabla_x\phi_0-c_2\nabla_y\phi_0\right)=0.
\end{aligned} \tag{S1.44}$$

We multiply equation (S1.27*b*) by c_0 and add this to equation (S1.43) to get,

$$\begin{aligned}
&\phi_0\left(\frac{\partial c_0}{\partial \tau_1}+\frac{\partial c_1}{\partial \tau_0}+\frac{\partial c_2}{\partial \tau_{-1}}\right)+\phi_1\left(\frac{\partial c_0}{\partial \tau_0}+\frac{\partial c_1}{\partial \tau_{-1}}\right)+\phi_2\frac{\partial c_0}{\partial \tau_{-1}}\\
&+\nabla_y\cdot(\boldsymbol{u}_1 c_0)+\boldsymbol{u}_0\cdot\nabla_y c_1+\nabla_x\cdot(c_0\boldsymbol{u}_0)\\
&-c_0\left(\frac{\partial \phi_0}{\partial \tau_1}+\frac{\partial \phi_1}{\partial \tau_0}+\frac{\partial \phi_2}{\partial \tau_{-1}}\right)-c_1\left(\frac{\partial \phi_0}{\partial \tau_0}+\frac{\partial \phi_1}{\partial \tau_{-1}}\right)-c_2\frac{\partial \phi_0}{\partial \tau_{-1}}\\
&-\nabla_y\cdot D\left(\phi_0\nabla_y c_2+\phi_0\nabla_x c_1+\phi_1\nabla_y c_1+\phi_1\nabla_x c_0\right)\\
&-\nabla_y\cdot D\left(\phi_2\nabla_y c_0-c_0\nabla_y\phi_2-c_0\nabla_x\phi_1-c_1\nabla_y\phi_1-c_1\nabla_x\phi_0-c_2\nabla_y\phi_0\right)\\
&-\nabla_x\cdot D\left(\phi_0\nabla_y c_1+\phi_0\nabla_x c_0+\phi_1\nabla_y c_0-c_0\nabla_y\phi_1-c_0\nabla_x\phi_0-c_1\nabla_y\phi_0\right)=0.
\end{aligned} \tag{S1.45}$$

We integrate (S1.45) over the domain B and apply the divergence theorem.

$$\begin{aligned}
&\int_B\left[\frac{\partial c_0}{\partial \tau_1}+\frac{\partial c_1}{\partial \tau_0}+\frac{\partial c_2}{\partial \tau_{-1}}+\nabla_x\cdot(c_0\boldsymbol{u}_0)\right]d\boldsymbol{y}+\int_{\partial B}\left[\hat{\boldsymbol{n}}\cdot(\boldsymbol{u}_1 c_0)+\boldsymbol{u}_0\cdot\hat{\boldsymbol{n}}c_1\right]d\boldsymbol{y}\\
&-\int_B\left[\nabla_x\cdot D\left(\phi_0\nabla_y c_1+\phi_0\nabla_x c_0+\phi_1\nabla_y c_0-c_0\nabla_y\phi_1-c_0\nabla_x\phi_0-c_1\nabla_y\phi_0\right)\right]d\boldsymbol{y}\\
&-D\int_{\partial B}\left[\hat{\boldsymbol{n}}\cdot\left(\phi_0\nabla_y c_2+\phi_0\nabla_x c_1+\phi_1\nabla_y c_1+\phi_1\nabla_x c_0+\phi_2\nabla_y c_0-c_0\nabla_y\phi_2\right)\right]d\boldsymbol{y}\\
&-D\int_{\partial B}\left[\hat{\boldsymbol{n}}\cdot\left(-c_0\nabla_x\phi_1-c_1\nabla_y\phi_1-c_1\nabla_x\phi_0-c_2\nabla_y\phi_0\right)\right]d\boldsymbol{y}=0,
\end{aligned} \tag{S1.46}$$

where we have used the knowledge that c_1 is independent of τ_{-1}, $\bar{C}$ and ϕ_1 are independent of τ_{-1} and τ_0, ϕ_0 is independent of τ_0, and ϕ_2 is independent of τ_{-1}. From the boundary condition, (S1.44), and equations (S1.3*a*) and (S1.28*a*), the boundary integrals in equation (S1.46) are zero.

Next, we average over the two faster time scales, τ_{-1} and τ_0. Firstly, we integrate (S1.46) with respect to τ_{-1} between 0 and $T_{-1} \gg 1$ such that ϕ_0 has been at steady state for sufficiently long time,

$$\begin{aligned}\int_B \left[c_2(T_{-1},\tau_0,\tau_1) - c_2(0,\tau_0,\tau_1)\right]\, d\boldsymbol{y} + T_{-1}\int_B \left[\frac{\partial c_1}{\partial \tau_0} + \frac{\partial c_0}{\partial \tau_1}\right]\, d\boldsymbol{y} \\ -T_{-1}\int_B \left[-\nabla_x \cdot (c_0\boldsymbol{u}_0) + \nabla_x \cdot D\left(\phi_0\nabla_y c_1 + \phi_0\nabla_x c_0 + \phi_1\nabla_y c_0\right)\right]\, d\boldsymbol{y} \\ +T_{-1}\int_B \left(c_0\nabla_y\phi_1 - c_0\nabla_x\phi_0 - c_1\nabla_y\phi_0\right)\, d\boldsymbol{y} = 0.\end{aligned} \tag{S1.47}$$

So that c_2 does not grow linearly with time, c_2 must be independent of τ_{-1}. Secondly, we integrate with respect to τ_0 between 0 and $T_0 \gg 1$ such that ϕ_0 has been at steady state for sufficiently long time

$$\begin{aligned}\int_B \left[c_1(\tau_{-1},T_0,\tau_1) - c_1(\tau_{-1},0,\tau_1)\right]\, d\boldsymbol{y} + T_0\int_B \left[\frac{\partial c_0}{\partial \tau_1} + \nabla_x\cdot(c_0\boldsymbol{u}_0)\right]\, d\boldsymbol{y} \\ - T_0\int_B \left[\nabla_x \cdot D\left(\phi_0\nabla_y c_1 + \phi_0\nabla_x c_0 + \phi_1\nabla_y c_0 - c_0\nabla_y\phi_1 - c_0\nabla_x\phi_0 - c_1\nabla_y\phi_0\right)\right]\, d\boldsymbol{y} = 0.\end{aligned} \tag{S1.48}$$

So that c_1 does not grow linearly with time c_1 must be independent of τ_0. We now substitute in series solutions for c_1 and $c_0 = \bar{C}\phi_0$,

$$\begin{aligned}\int_B \Bigg[\frac{\partial c_0}{\partial \tau_1} + \nabla_x\cdot\left(\bar{C}\phi_0\boldsymbol{u}_0\right) \\ -\nabla_x\cdot D\left(\phi_0\nabla_y\left(\xi_k^c\partial_{x_k}\bar{C}\right) + \phi_0^2\nabla_x\bar{C} - \left(\xi_k^c\partial_{x_k}\bar{C}\right)\nabla_y\phi_0\right)\Bigg]\, d\boldsymbol{y} = 0.\end{aligned} \tag{S1.49}$$

Terms containing ϕ_1 are neglected as they are order λ^2. So, by substituting in the series solution for $\boldsymbol{u}_0$,

$$\begin{aligned}\|B\|\left(S\frac{\partial\bar{C}}{\partial\tau_1} + \bar{C}\frac{\partial S}{\partial \tau_1}\right) + \nabla_x\cdot\left(\bar{C}\boldsymbol{U}\right) \\ - \nabla_x\cdot D\left(\int_B \left[\phi_0^2 I + \left(\phi_0\nabla_y\xi_k^c - \xi_k^c\nabla_y\phi_0\right)\otimes\hat{\boldsymbol{e}}_k\right]\, d\boldsymbol{y}\nabla_x\bar{C}\right) = 0.\end{aligned} \tag{S1.50}$$

where I is the identity matrix. Note that,

$$\int_{-\infty}^{\infty}\phi_0^2\, d\boldsymbol{y} - \int_{-\infty}^{\infty}\phi_0\, d\boldsymbol{y} \sim \mathcal{O}(\lambda), \tag{S1.51}$$

therefore $\int_B \phi_0^2\, d\boldsymbol{y} \to \int_B \phi_0\, d\boldsymbol{y}$ as $\lambda \to 0$. Hence, we can write,

$$\|B\|\left(S\frac{\partial\bar{C}}{\partial\tau_1} + \bar{C}\frac{\partial S}{\partial\tau_1}\right) + \nabla_x\cdot\left(\bar{C}\boldsymbol{U}\right) - \nabla_x\cdot D\left(\|B\|S\boldsymbol{I} + D_{eff}\right)\nabla_x\bar{C} = 0, \tag{S1.52a}$$

where

$$D_{eff} = \int_B \left(\phi_0\nabla_y\xi_k^c - \xi_k^c\nabla_y\phi_0\right)\otimes\hat{\boldsymbol{e}}_k\, d\boldsymbol{y}. \tag{S1.52b}$$

The final macroscale equations are,

$$\|B\|\frac{\partial S}{\partial \tau_1}+\nabla_x\cdot\boldsymbol{U}=0, \qquad \boldsymbol{x}\in\Omega, \tag{S1.53a}$$

$$\nabla_x\cdot\bar{\boldsymbol{U}}=0, \qquad \boldsymbol{x}\in\Omega, \tag{S1.53b}$$

$$\|B\|\left(S\frac{\partial \bar{C}}{\partial \tau_1}+\bar{C}\frac{\partial S}{\partial \tau_1}\right)+\nabla_x\cdot\left(\bar{C}\boldsymbol{U}\right)-\nabla_x\cdot D\left(\|B\|S\boldsymbol{I}+D_{eff}\right)\nabla_x\bar{C}=0, \qquad \boldsymbol{x}\in\Omega, \tag{S1.53c}$$

where

$$\boldsymbol{U}=\frac{K[S,\theta(\bar{C})]}{\eta(\bar{C})}\nabla_x\mu_0+\frac{b[S,\theta(\bar{C})]}{\eta(\bar{C})}\nabla_x p_0+\frac{b_g[S,\theta(\bar{C})]}{\eta(\bar{C})}\hat{\boldsymbol{e}}_3 g, \tag{S1.53d}$$

$$\bar{\boldsymbol{U}}=\frac{\bar{K}[S,\theta(\bar{C})]}{\eta(\bar{C})}\nabla_x\mu_0+\frac{\bar{b}[S,\theta(\bar{C})]}{\eta(\bar{C})}\nabla_x p_0+\frac{\bar{b}_g[S,\theta(\bar{C})]}{\eta(\bar{C})}\hat{\boldsymbol{e}}_3 g, \tag{S1.53e}$$

and

$$\mu_0=F[S,\theta(\bar{C})]\gamma(\bar{C}), \tag{S1.53f}$$

and the saturation dependent parameters are given by equations (S1.39), (S1.42) and (S1.52*b*).

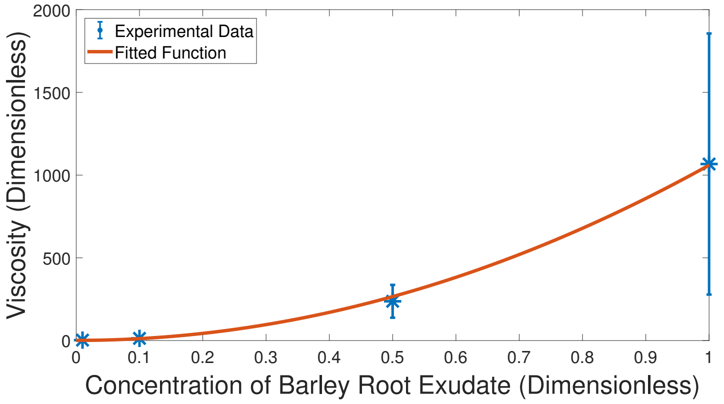

Figure S1.1: Experimental measurment of the viscosity of barley root exudate at different concentrations. The fitted line is $1059\bar{C}^2 + 1$.

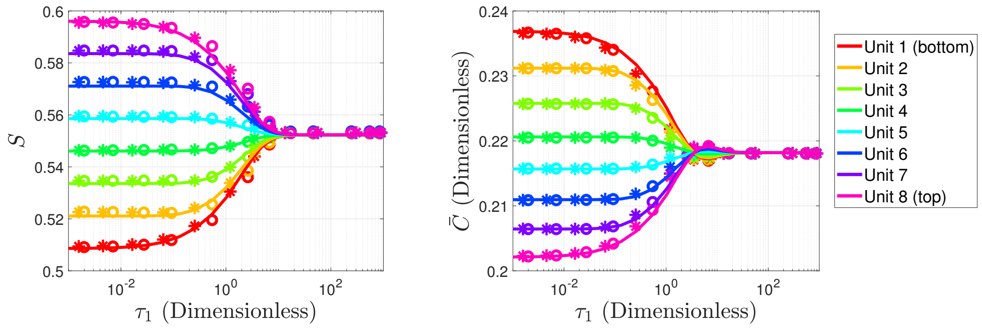

(a) Saturation (b) Concentration

Figure S1.2: Comparison of full model with original viscosity (*), full model with assumed viscosity (o) and homogenised model (solid line) for a small perturbation that does not include a Haines' jump. The colours represent the different geometry units in the soil column. S is the saturation, $\bar{C}$ is the macroscale concentration and τ_1 is the slow time scale.
